# Genomics and Machine Learning for Taxonomy Consensus: The *Mycobacterium tuberculosis* Complex Paradigm

**DOI:** 10.1371/journal.pone.0130912

**Published:** 2015-07-08

**Authors:** Jérôme Azé, Christophe Sola, Jian Zhang, Florian Lafosse-Marin, Memona Yasmin, Rubina Siddiqui, Kristin Kremer, Dick van Soolingen, Guislaine Refrégier

**Affiliations:** 1 LIRMM UM CNRS, UMR 5506, 860 rue de St Priest, 34095 Montpellier cedex 5, France; 2 Institute for Integrative Biology of the Cell (I2BC), CEA, CNRS, Université Paris-Sud, rue Gregor Mendel, Bât 400, 91405 Orsay cedex, France; 3 Pakistan Institute for Engineering and Applied Sciences (PIEAS), Lehtrar Road, Nilore, Islamabad, Pakistan; 4 Health Biotechnology Division, National Institute for Biotechnology and Genetic Engineering (NIBGE), P.O. Box # 577, Jhang Road, Faisalabad, Pakistan; 5 National Institute for Public Health and the Environment, P.O. Box 1, 3720 BA Bilthoven, The Netherlands; 6 Department of Pulmonary Diseases and Department of Microbiology, Radbout University Nijmegen Medical Centre, University Lung Centre Dekkerswald, P.O. Box 9101, 6500 HB Nijmegen, The Netherlands; University of Brighton, UNITED KINGDOM

## Abstract

Infra-species taxonomy is a prerequisite to compare features such as virulence in different pathogen lineages. *Mycobacterium tuberculosis* complex taxonomy has rapidly evolved in the last 20 years through intensive clinical isolation, advances in sequencing and in the description of fast-evolving loci (CRISPR and MIRU-VNTR). On-line tools to describe new isolates have been set up based on known diversity either on CRISPRs (also known as spoligotypes) or on MIRU-VNTR profiles. The underlying taxonomies are largely concordant but use different names and offer different depths. The objectives of this study were 1) to explicit the consensus that exists between the alternative taxonomies, and 2) to provide an on-line tool to ease classification of new isolates. Genotyping (24-VNTR, 43-spacers spoligotypes, IS*6110*-RFLP) was undertaken for 3,454 clinical isolates from the Netherlands (2004-2008). The resulting database was enlarged with African isolates to include most human tuberculosis diversity. Assignations were obtained using TB-Lineage, MIRU-VNTR*Plus*, SITVITWEB and an algorithm from Borile *et al*. By identifying the recurrent concordances between the alternative taxonomies, we proposed a consensus including 22 sublineages. Original and consensus assignations of the all isolates from the database were subsequently implemented into an ensemble learning approach based on Machine Learning tool Weka to derive a classification scheme. All assignations were reproduced with very good sensibilities and specificities. When applied to independent datasets, it was able to suggest new sublineages such as pseudo-Beijing. This Lineage Prediction tool, efficient on 15-MIRU, 24-VNTR and spoligotype data is available on the web interface “TBminer.” Another section of this website helps summarizing key molecular epidemiological data, easing tuberculosis surveillance. Altogether, we successfully used Machine Learning on a large dataset to set up and make available the first consensual taxonomy for human *Mycobacterium tuberculosis* complex. Additional developments using SNPs will help stabilizing it.

## Introduction

Bacterial taxonomy has logically emerged when technology unveiled the microscopic level of life at the end of the nineteenth century. Macroscopic organisms were being classified since Aristotle, 350 BC [1,2], and the same taxonomic levels (Species, Genus, Family, Order, Class, Phylum, and Reign) were chosen. The process of assigning an organism inside a definite taxonomy will thereafter be referred to as classification, the term taxonomies being used for the classification schemes themselves. For the Species level, microbiologists have applied various concepts of species definition (phenotypical, morphological, ecological), trying to identify groups of organisms with identical biochemical features, studying colony morphology, nutrition requirements, etc., as well as host symptoms for pathogenic species. The similarities between lineages were randomly or intuitively ordered. With the advent of molecular biology, DNA-DNA hybridization has been recognized as a powerful, objective and consistent tool for characterizing lineages, and the threshold of 70% identity has been proposed for species delineation [1]. 16S rRNA sequence has then attracted attention: the presence of conserved regions ensured amplification in any bacteria. 16S rRNA sequences retrieved environmental samples helped describe bacterial diversity among uncultivable organisms. The comparison between DNA-DNA hybridization data with 16S rRNA sequences has shown that organisms with less than 97% identity in 16S rRNA sequence could safely be considered as belonging to different species [3]. Whole Genome sequences of Bacteria are now easily acquired due to their relative small size (below 10 Mb). The Average Nucleotide Identity (ANI) assesses DNA identity between two genomes and proves very concordant with DNA-DNA Hybridization. A threshold of 95% ANI is now advised to name new species [4]: individuals with more than 95% ANI should be considered as belonging to the same species.

Tuberculosis agent was first isolated by Robert Koch in 1882, the name *Bacillus tuberculosis* was proposed to the community by Zopf in 1883, which was changed for *Mycobacterium tuberculosis* in 1907 [5]. In 1912, a bacillus isolated by E. Nocard on cows and today known as *M*. *bovis* was specifically used to set up a vaccine after serial passage experiments led by Calmette and Guérin; it did not receive a different species name at that time, possibly because it was known to infect humans and trigger symptoms very similar to those of *M*. *tuberculosis*. In contrast, a bacillus collected on rodents, initially referred to as *M*. *tuberculosis* var. *muris*, was officially raised to the species level in 1957 [6] in a period where scientists promoted the use of infectivity profile as a mean of pathogen characterization [7]. Following this rationale, an increasing number of species were named according to their principal animal host even if alternative mammal hosts were common and disregarding DNA similarity criteria: *M*. *bovis* in 1970, *M*. *caprae* in 1999, *M*. *pinnipedii* in 2003, *M*. *mungi* in 2010, *M*. *orygis* in 2012, *M*. *surricatae* in 2013 [8,9,10,11,12,13]. Two lineages infecting humans were raised to the level of species because of specific metabolic and phenotypic features: *M*. *africanum* and *M*. *canettii* [14,15]. In fact, the DNA diversity within and between all these lineages as studied by Whole Genome Sequencing proved limited, except for *M*. *canettii*’s showing higher intra- and inter- lineage diversity [16,17,18]. According to molecular data such as average nucleotide identity (ANI) and 16S rRNA divergence, all these “species” could be considered as a single one despite the clear diversity in host spectrum. In contrast, some *M*. *tuberculosis* isolates proved more distant from one another than one was from any animal isolates. The resemblance between all the species listed above has led to the advent of “*M*. *tuberculosis* complex” (MTC) terminology for more than 30 years [19]. The diversity among human isolates harboring similar metabolic features being higher than the diversity between animal isolates whatever the host species [20], infra-species taxonomy of *M*. *tuberculosis* affecting humans is as important as the description of animal isolates diversity.

RFLP data detecting *IS*6110 insertion sequence and/or that of CRISPR locus (long called “Direct Repeat” or “DR locus”, and the method, “Spoligotyping”) have initiated MTC infra-species MTC taxonomies strictly based on genotyping data [21,22,23]. The taxonomy based on CRISPR locus, relying on the presence/absence of specific spacers, became soon the most extensively used and a worldwide database referred to as SpolDB and later SITVITWEB is including an increasing number of sublineages since 1999 [24,25,26]. The CRISPR locus was found to have carried 68 spacers in the most recent common ancestor to all MTC species except *M*. *canettii* [27,28] and to have subsequently evolved by the loss of spacers or the integration of *IS*6110 sequences [29]. Recurrent “signatures”, *i*.*e*. the absence of specific spacers, easily detected by experts, led to the naming of LAM, CAS, S, X, etc. sublineages [30]. The relevance of the corresponding taxonomy has been promptly acknowledged by the tuberculosis community based on the good congruence with geographical data, previously described ecological species *M*. *africanum*, *M*. *bovis*, sublineages such as Beijing and Haarlem detected using *IS*6110-RFLP [31,32]. Other studies criticized this taxonomy based on the fact that the deletion of each spacer considered independently can suffer from convergence [33,34]. However, these criticisms were defeated for major signatures as defined in SITVITWEB [35,36]. The reason for the reliability of well-known signatures despite convergence effects on individual spacers is that these signatures take into account the spatial organization of the locus. In the end, CRISPR-derived taxonomy is still widely used with 100 hits in Pubmed during the last 12 months (as assessed on February 12^th^, 2015) using keywords “(spoligo* OR CRISPR) AND tuberculosis”. The automation of CRISPR data use for classification has led to several web interfaces: *spoligoforest* to infer transmission chains SPOTCLUST and TB-Lineage for labeling new isolates [29,37,38], with TB-Lineage using a simplified taxonomy indicating large lineages as defined by Gagneux *et al*. [38,39].

From 2000 on, tuberculosis taxonomy was complexified by the advent of large deletions [40,41] and minisatellites termed MIRU-VNTR (mycobacterial interspersed repetitive units, variable number tandem repeats) [42,43,44,45,46,47]. The large acquisition of MIRU-VNTR data soon suggested that at least some specific labels provided using spoligotype patterns could be flawed [48]. An independent database and the corresponding taxonomy was set up to classify isolates according to the standardized combination of 24 MIRU-VNTR patterns [49], and/or Regions of deletion: MIRU-VNTR*Plus* [47,50]. These 24-VNTR genotypes can be used in parallel for molecular epidemiology as they include many loci with high discriminatory power [51,52,53]. The most variable loci form a 15-MIRU-VNTR set that is now collected for epidemiological surveillance by most Reference labs, in combination or not with spoligotyping [54].

The next step in MTC diversity exploration was the analysis of Single Nucleotide Polymorphisms (SNP) either using high-throughput SNP typing [55,56,57] or Whole Genome Sequencing [39,58,59,60,61,62,63]. These approaches largely validated spoligotype and MIRU-VNTR based taxonomies [34]. Several studies propose new classification tools using SNPs detection, the most precise and consensual being that derived from the data mining in more than 1,000 genomes [62].

Altogether, several tools currently exist for assigning *M*. *tuberculosis* complex isolates to taxonomic groups at different depths, but little time was by now invested in characterizing the concordance between the corresponding taxonomies. As a consequence, TB epidemiologists are often puzzled when trying to characterize their isolates. There is a clear need for using large studies and powerful algorithms on large datasets for establishing consensual infra-specific MTC taxonomy.

Machine learning is a statistics science identifying relevant information in large datasets even when some data are missing. It involves pattern recognition in prototypes *i*.*e*. the identification of formal rules correlated to a characteristic of interest [64]. Once patterns have been identified, assignation of unknown individuals is easy and very fast. Such method has been applied previously on spoligotyping data and helped identifying informative spacers to recognize experts-based groups [65]. Weka is a work bench set up in 1997 and implementing state of the art machine learning algorithms. This ability proved critical for reducing assignation errors [66]. It is very popular and was used in studies as diverse as epilepsy characterization using imaging data [67] and methylated DNA patterns linked to genetic diseases [68]. Its swiftness enables to implement it in parallel on different type of data, so that complete annotation of very large dataset can be reached in a timeframe of one minute.

In this work, we first completed the genotyping of a large dataset of 3,454 human *M*. *tuberculosis* isolates from the National Reference Center of Netherlands (RIVM) collected between 2004 and 2008 [69]. This data was used to further describe TB diversity and transmission dynamics in Netherlands and to clarify the potential of spoligotyping in molecular epidemiology. We then used this large database as a reference for human *M*. *tuberculosis* worldwide diversity after complementing it with genotypes from Lineages 5 and 6 particularly underrepresented in the RIVM dataset. We annotated all these genotypes according to existing classification tools to search for stable correspondences between the underlying taxonomies and proposed a new consensus where the correspondences are made explicit. We finally used Weka software to learn in parallel classification procedures, handling Spoligotype or MIRU-VNTR data, reproducing original and new taxonomies, and made the most successful procedure available on-line. Altogether, our work successfully clarifies the correspondence between the existing *M*. *tuberculosis* complex taxonomies. This consensus can be retrieved for any sufficiently informed new genotype (best when including at least Spoligotype + 15 MIRU-VNTR) using our new web interface, TBminer.

## Material and Methods

### Isolates

All isolates analyzed in this study were cultured on Lowenstein Jensen solid media or 7H9 liquid medium in MGIT960 device. Three thousand four hundred and fifty four (n = 3,454) were from the National Reference Center of Netherlands, also committed in worldwide quality control studies. 225 isolates were from diverse hospitals in Pakistan (Faisalabad n = 6; Islamabad National Reference Center n = 109; Karachi n = 29; Lahore n = 21; Rawalpindi n = 60). DNA was extracted using the standard procedure using Cetyl-trimethyl-ammonium bromide (CTAB) [70]. No information concerning the patients was included in the analysis so that no approval from any ethical comity was required.

### High throughput Luminex-based spoligotyping

A total of 3,454 DNA samples, extracted from isolates collected between 2004 and 2008 by the RIVM and sent as concentrated CTAB (Cetyl-trimetthyl-ammonium bromide) extracts, were genotyped by high-throughput Luminex spoligotyping [23,27,71,72]. Briefly, 1 μL of ~50 ng/μL DNAs were amplified by PCR using DRb and biotinylated DRa in 25μL. PCR product (2μL) were hybridized with coupled polystyrene microbeads (2500 Microplex microbeads per individual target, Luminex Corp, Austin, USA) for 30mn at 55°C in 1.5X TMAC (Tetra-methyl ammonium chloride). After washing, 2μL streptavidin-R-phycoerythrin (1mg/mL, Invitrogen, USA) was added, microbeads were centrifuged and washed again, and after resuspending in 1x TMAC, the plates were read at 52°C. Interpretation was made using home-made excel matrixes helping control of cut-off selection between positive and negative values for each spacer. A Quality Control check, done by an independent investigator on 5% of randomly selected samples showed a perfect reproducibility of the patterns.

Twenty-two (22) samples could not be genotyped by spoligotyping totaling 3432 fully genotyped isolates (complete spoligotype, IS*6110*-RFLP and maximum 1 out of 24 MIRU-VNTR missing).

### Assignation to sublineages using available classifications

Files of 500 24-VNTR and spoligotyping genotypes complying with all specified requirements were loaded onto TB-lineage and MIRU-VNTR*Plus* websites. In MIRU-VNTR*Plus*, default settings were changed to assign isolates to the closest inside the curated database as soon as the distance is of 0.5 maximum (default = 0.17), so that most isolates could be classified (n = 3382). TB-lineage could classify 3283 isolates (i.e. 97%).

To assign genotypes according to SITVITWEB taxonomy, we first made use of an Excel version of SpolDB4 uncovering the 2881 first SITs implemented in SITVITWEB. The assignations were slightly corrected by taking into account recent knowledge on relatedness among Euro-American sublineages. For instance, CAMEROON genotypes previously referred to as LAM10_CAM and subsequently found not to be related to LAM were simply labeled CAM, TURKISH isolates previously referred to as LAM7_TUR and subsequently found not to be related to LAM were simply labeled TUR, H4 subsequently found not to be related to Haarlem were renamed URAL, and more precisely URAL1 when spacer 2 was present and URAL2 when spacer 2 was absent. For the unclassified isolates, we used expert knowledge of C. Sola, mainly applying rules as described in Filliol *et al*.[73,74].

To assign each genotype according to Borile *et al*. taxonomy, we computed distances to the 32 Borile references based on shared blocks of absent spacers [36]. Every isolates was assigned to the group of the most similar reference, and unassigned when equal distances were found with at least 2 references.

### Taxonomy consensus identification

Correspondences between all taxonomies were listed using an in-house algorithm to identify synonyms. As 24-VNTR signatures are known to be less prone to convergence than deletions of individual spacers in spoligotype patterns [34], when assignations by SITVITWEB and MIRU-VNTR*Plus* taxonomies were conflictive, we privileged MIRU-VNTR*Plus* assignation. To make the synonym explicit, we tended to concatenate short versions of the different synonyms unless it was too long (Table 1).

**Table 1:** Correspondence table between the different *M*. *tuberculosis* taxonomies.

TBlineage	MIRU-VNTR*Plus*	SITVITWEB	Borile-AP	Consensus Expert	*Coll et al*.
Lineage 6	West African 2	AFRI_1	Afri1	**L6_Afri1**	*6*
(West African 2)		AFRI			
Lineage 5	West African 1	AFRI_2	Afri2-3	**L5_Afri2**	*5*
(West African 1)		AFRI_3			
Animal strains	Bovis	BOVIS	bovis	**L0_Animal**	*M*. *bovis*
		MICROTII, PINI	Pin-Mic		
		CAP	Cap		
Lineage 1 (Indo Oceanic)	EAI	EAI1_SOM	EAI1	**L1_EAI1**	*1*.*2*.*2*
		EAI2_MANILLA,NTB	EAI2	**L1_EAI2**	*1*.*2*.*1*
		EAI3_IND	EAI3-5	**L1_EAI3**	*1*.*1*.*2*
		EAI4_VNM	EAI	**L1_EAI**	*1 & 1*.*1*.*1*
		& EAI5			
		EAI6, EAI 7	EAI6	**L1_EAI6**	*1*.*1*.*3*
Lineage 2 (East Asia)	Beijing	BEIJING	Beij	**L2_Beijing**	*2*.*2*
		BEIJING-LIKE			
Lineage 3	Dehli/CAS	CAS1_DEHLI	CAS	**L3_CAS**	*3*
(India and		CAS1_KILI			*(3*.*1*.*1)*
East Africa)		CAS2			*(3*.*1*.*2)*
		*BEIJING*	*Beijing*		*(-)*
Lineage 4	Ghana	T1	T1a—T1b—T1c	in L4	*in 4*
(Euro-	UgandaI-II	T2, T2_UGANDA	T2	**L4_Uganda(T2)**	*4*.*6*.*1*
American)		EAST_MED1	T(T1-H-CAM)	in L4	*4*.*6* *?*
		LAM3_S			
	?	T3	?	in L4	*4*.*6*.*2* *?*
	?	T4_CEU	T4	**L4_T4**	*4*.*8* *?*
	?	T5_MAD2	T5	**L4_T5**	*4*.*7a* *?*
	H37Rv	H37Rv	?	**L4_H37Rv**	*4*.*9*
	TUR	LAM7_TUR	?	**L4_TUR**	*4*.*2*.*2*
		T1	?		
	URAL	H4 (remaned Ural1)	Ural	**L4_Ural1**	*4*.*2*.*1a*
	New-1	H4 (remaned Ural2)	?	**L4_Ural2(New1)**	*4*.*2*.*1b*
	S	S	S	**L4_S**	*4*.*4*.*1*
	Cameroon	LAM10_CAM	T-T1	**L4_CAM**	*4*.*6*.*2*
	Haarlem	H1, H2	H1-2	**L4_H1-2**	*4*.*1*.*2*
		H3, H3-T3	H3	**L4_H3**	*4*.*5* *?*
	LAM	LAM1, LAM2, LAM5	LAM5-2-1	**L4_LAM**	*4*.*3*
		LAM3, 4, 6, 8	LAM3		
		LAM9, 11, 12	LAM9-11		
		T5_RUS1	T(T1-H-CAM)		
		T5	?	**L4**	*4*.*7b* *?*
	X	X2	X2	**L4_X**	*4*.*1*.*1*
		H1	H1-2		*4*.*1*
	X, Haarlem	X1, X3	X1-3		*4*.*1*
?	?	MANU1, MANU2, ZERO	Manu	**?**	*2*.*1* *?*

The items in italics were subsequently added according to findings in complementary analyses. Parts under brackets indicate synonyms. Complete sublineages under brackets indicate imprecise correspondance. “?” indicate hypotheses with no actual proof.

### Classification learning using Weka

A curated database enlarged to include more *M*. *africanum* isolates was imported into Weka. More specifically, curation removed all isolates for which at least one taxonomy was not able to provide an assignation or when one VNTR had a zero value (indeed absence of results was at some point in this long-lasting study recorded as 0 which may have lead to erroneous profiles); this concerned all lineages (data not shown) and almost all MIRU-VNTR so that it is not likely to have introduced any bias in the database. *M*. *africanum* isolates added to the RIVM curated dataset came from a Nigerian study. Altogether, the database counted 2,904 isolates including at least 36 isolates of main human tuberculosis lineages: 36 L0_*M*. *bovis*, 313 L1_EAI, 213 L2_Beijing, 270 L3_CAS, 1975 L4_Euro-American, 58 L5_Waf1-Afri2, 36 L6_Waf2-Afri1.

Weka was used to train a classifier using different Machine learning algorithms and a Vote procedure. Machine algorithms handle characteristics such as genotype patterns at different loci, and a selected feature to be predicted. Every genotypic characteristic handled independently is called an “attribute”. Different algorithms exist. A short description of their different characteristics is as follows: 1) **J48** algorithm is the Weka version of the C4.5 algorithm. J48 is used to learn decision trees using quantitative values of attributes. First, the pair (attribute, value) that optimizes a criterion (entropy, gini index) is used to split the data in two sample. Then, for each sample, if it is pure (only one lineage represented) or if it contains less than a predefine number of data, the tree growing is stopped, else another split is determined based on the same algorithm; 2) **JRip** algorithm (with Rip standing for Repeated Incremental Pruning, and J for Java, the programming language used to implement Weka) an algorithm already implemented on tuberculosis data for TB-lineage [38] consists in identifying an ordered list of complex rules using quantitative values, beginning with less prevalent class; if the first set of rules is fulfilled, the isolate is assigned to the corresponding lineage, otherwise the next set of rules is examined; a default assignation is proposed at the end of the list if no set of rules has been fulfilled; 3) **Naive Bayes** algorithm is based on the assumption that attributes are independent. Despite the fact that this assumption is rarely true, the obtained classifier has frequently good performances. The main idea of Naive Bayes is to determine the lineage maximizing the probability of being associated to a given set of attributes (here spacers in spoligotype pattern, individual MIRU-VNTRs). This probability is the product of all the marginal probabilities of each attribute associated to the lineage (this product makes sense only under the assumption of attributes independence); **PART** uses rules in the same way JRip does, but these rules are built using a decision tree as does J48; **Random Forest** consists in assigning each isolates using a multitude of decision trees and provides the assignation that is the mode among all of these trees; the decision trees are built partly randomly. All these methods can undergo meta-bagging procedures to reduce the impact of overfit to the data. Overfitting occurs when lots of data are used to set up the classifiers so that some irrelevant features are included in the decision trees or rules. Meta-bagging consists in randomly selecting features and data used to induce the classifiers, a step that actually reduces the chances for learning irrelevant features.

All algorithms can be combined and contribute to a Vote step during which the most frequent assignation is selected.

Evaluation was performed for each algorithm using stratified cross-validation. Stratified cross-validation consists in partitioning the training dataset in a specified number of folds k, learn on k-1 folds and test the algorithm of the remaining fold. The number of folds was varied between 3 and n-1 (leave-one-out procedure, n corresponding to the total number of items). When the number of folds is low, the computed accuracy is more likely to match that obtained on an independent dataset as the testing set is quite large, however, the rules or trees inferred may be less precise as they have been set up with smaller training data.

The “Vote-10” algorithm (using all 5 original algorithms as well as all their 5 meta-bagging derivates) was applied on the complete training dataset to build up the Lineage prediction classifier available on TBminer website.

### Datasets for independent evaluation of Lineage Prediction tool

The first independent dataset was that of MIRU-VNTR*Plus* database uncovering 186 isolates from both human and animal tuberculosis.

The second independent dataset was built by complete genotyping as described in Yasmin *et al*. [53]. Only isolates with “complete” spoligotype and 24-MIRU-VNTR genotypes i.e. lacking at most one VNTR (n = 225) were kept. Isolates were mainly from Punjab. Altogether, only 38 datapoints were missing out of 15,075, mostly from VNTR Qub26 (n = 10), Qub11b (n = 7) and ETR-A (n = 6).

## Results

### Characteristics of the RIVM dataset and contribution of spoligotyping to molecular epidemiology

**Fig 1:**
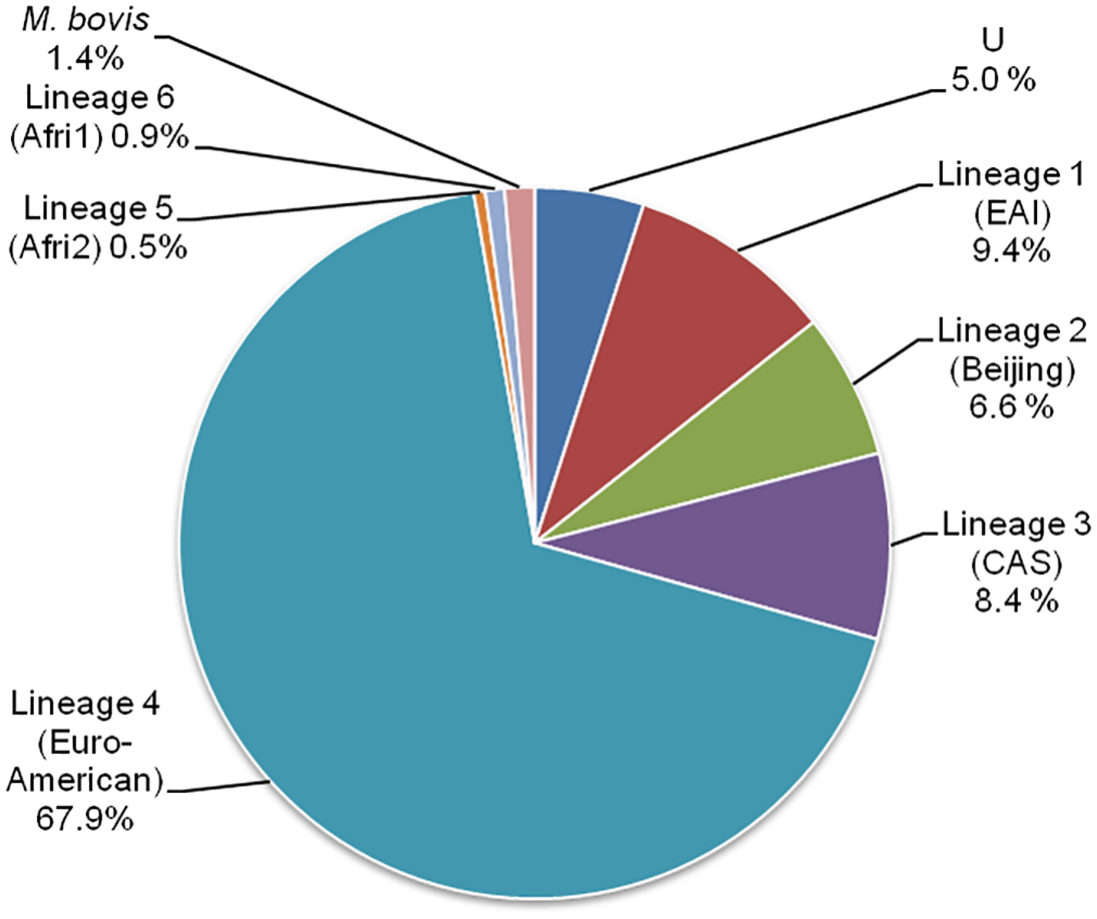
Relative prevalence of main *M*. *tuberculosis* complex lineages in the Netherlands (2005–2008).

We characterized by spoligotyping 3,454 DNA samples retrieved from as many clinical isolates of the RIVM collection (2004–2008). Genotypes obtained by 24-MIRU-VNTR and IS*6110* RFLP profiles were already available [69]. Ninety-nine percent of the patterns were successfully obtained (99.4%; n = 3432). According to TB incidence estimates in the Netherlands for this period (n = 1330 per year as estimated in 2004), this represents around 65% of all TB cases [75]. Assignation of genotypic profiles to described lineages was performed independently for each isolate and for each taxonomy using available on-line tools: 1) SITVITWEB database using spoligotype patterns and upgraded by the expert eye for new profiles, 2) MIRU-VNTR*Plus* assignation tool implementing similarities on 24 MIRU-VNTR patterns, 3) TB-Lineage using both spoligotype patterns and 24 MIRU-VNTR information, 4) Borile *et al*.-derived in-house algorithm using spoligotype profiles only [36]. The resulting file is available as S1 Table **.** Alternative taxonomies were largely concordant as detailed below. Most prevalent lineage according to TB-Lineage taxonomy was the so-called “lineage 4” (also referred to as Euro-American lineage, 68%, n = 2,345). Lineages 1, 2 and 3 (corresponding to EAI, Beijing and CAS SITVITWEB denominations) corresponded each to slightly less than 10% of the isolates (n = 324, 228 and 291 respectively), lineages 5 and 6 (designated as Afri2 and Afri1 in SITVITWEB) and *M*. *bovis* lineage representing around 1% of the isolates each (n = 30, 18 and 47 respectively, Fig 1). Among the 171 isolates (5.0%) that could not be classified using TB-lineage, most of them were classified by MIRU-VNTR*Plus* as being part of Euro-American lineage (Haarlem, n = 50) or EAI (n = 23). Altogether, this RIVM dataset is typical of that of a Western country (predominance of Euro-American lineage) with worldwide immigration providing larger diversity. Euro-American isolates infra-lineage diversity (67.9% of the total dataset) can be described using the MIRU-VNTR*Plus* classification. Most were labeled Haarlem (n = 777; 22.5% of total dataset), second, LAM (n = 548; 15.9%) and third, Cameroon (n = 260; 7.5%).

We assessed the clustering level in this database and set up an online tool to automate it, available at info-demo.lirmm.fr/TBminer/ (Clusters Analysis option). We used 24-VNTR profiles to define clusters as it stands as the current Gold standard for molecular epidemiology since 2006 [49,69,76]. We detected 109 to 295 clustered isolates each year (S2 Table). When considering the global 5-years set, the number of clustered isolates reached 1,362 which was higher than the sum of clustered isolates for each separate year. This indicates that unique isolates collecting during different years carry the same genotype. Such phenomenon may illustrate the likely underestimation of clustered cases if performing smaller studies over a shorter period as previously shown theoretically [77]. Alternatively, it may be caused by a lack of discriminatory power in the genotyping techniques, as recently shown by Whole Genome Sequencing [78]. Recent Transmission Index as computed using the “n-1” method [79] varied from 10.8% to 26.6% when considering each year separately but reached 29% when considering all 5-years sampling period (S2 Table). Mean cluster size was 3.8 isolates but one cluster belonging to LAM lineage was made of 64 isolates and nineteen clusters exhibited more than 10 isolates. We explored if these clusters could be split when considering spoligotyping data (Table 2). All spoligotype patterns of isolates belonging to the same 24-VNTR cluster were similar. The isolates carrying different spoligotype signatures often were collected on different years. Inside these 24-VNTR clusters, spoligotype patterns harboring more deletions were rather posterior to patterns with fewer deletions (n = 21), albeit with several exceptions (n = 7; Table 2). This confirms that both rare mutations in spoligotype pattern without changes in the 24-VNTR set may occur, and that convergence in 24-VNTR data for related isolates is not so rare. On the whole dataset, adding spoligotyping to 24-VNTR typing refined clustering by 10.1% (S1 Fig), likely identifying most convergence events among 24-VNTR clusters that could be detected using Whole Genome data [59].

**Table 2:** Major clusters of the 2004–2008 Netherlands RIVM collection (n≥10).

24-VNTR Cluster ID	n	Different spoligotype patterns within the same 24-VNTR cluster	SIT	Sublineages (SITVITWEB classification)	Year of first isolation	ID of eldest isolates with corresponding genotype
		νν○ννννννννννννννννν○○○○νννννννν○○○○ννννννν	20		2004	NLA000400263
1	64	νν○ννννν○ννννννννννν○○○○νννννννν○○○○ννν○ννν	New	LAM 1	2005	NLA000500735
		νν○ν○ννννννννννννννν○○○○νννννννν○○○○ννννννν	729		2006	NLA000601675
		ννννννννννννννννννννννννν○○○○○○ν○○○○ννν○ννν	62	H1	2004	NLA000400246
2	53	ννννννννννννννννννννννννν○○○○○○ν○○○○ννννννν	47		2005	NLA000500437
		○○○○○○○○○○○○○○○○○○○○○○○○○○○○○○○○○○○○○○○○○○○	2669	U	2006	NLA000600009
3	33	ννννννν○○○νννννννννννννννννννννν○○○○ννν○ννν	New	U	2004	NLA000400201
4	28	ννννννννννννννννννν○νννννννννννν○○○○ννν○ννν	736	T2	2004	NLA000400425
		ννννννννννννννννννν○○○○○○ννννννν○○○○ννν○ννν	New		2005	NLA000501826
5	22	νννννννννννννννννννν○○○○νννννννν○○○○ννννννν	42	LAM9	2004	NLA000400150
		νννννννννννννννννννννννννννννννν○○○○ννννννν	53	T	2005	NLA000500775
6	19	ννν○○○○νν○ννννννννν○○○○○○○○○○○○○○○○νννννννν	21	CAS1_KILI	2004	NLA000401265
		ννν○○○○νννννννννννν○○○○○○○○○○○○○○○○νννννννν	22		2005	NLA000500746
		νννννννννννν○νννννν○νν○ννννννννν○○○○ννννννν	1227		2004	NLA000400237
7	18	ννννννννννννννννννν○νν○ννννννννν○○○○ννννννν	58	T5_MAD2	2004	NLA000400972
		νννννννννννννννννννννν○ννννννννν○○○○ννννννν	44		2004	NLA000401032
8	17	νν○νννν○○○○○○○○○○○○○○○○○○ννν○○○○ν○ννννννννν	89	EAI2_NTB	2004	NLA000400077
9	15	ννννννννννννννννννννννννν○ννν○νν○○○○ννννννν	1558	T1	2004	NLA000400231
10	14	○○○○○○○○○○○○○○○○○○○○○○○○ν○○○○○○ν○○○○ννννννν	2	H2	2004	NLA000400112
		ννννννννννννννννννν○○○○○ν○○ννννν○○○○ννννννν	41		2004	NLA000401211
		νν○νννννννννννννννν○○○○○ν○○ννννν○○○○ννννννν	930		2005	NLA000500774
11	14	ννννννννννννννννννν○○○○○ν○○ννννν○○○○ννν○ννν	1261	TUR	2005	NLA000501593
		νννννννννννν○νννννν○○○○○ν○○ννννν○○○○ννννννν	367		2006	NLA000601569
		ννν○○νννννννννννννν○○○○○ν○○ννννν○○○○ννννννν	New		2007	NLA000701171
		ννν○○○○ννννννννννννννν○○○○○○○○○○○○○νννννννν	203		2004	NLA000401787
12	13	ννν○○○○ννννννννννννννν○○○○○○○○○○ν○○νννννννν	New	CAS	2005	NLA000500783
		ννν○○○○νννννννννννννν○○○○○○○○○○○○○○○ννννννν	1949		2008	NLA000800421
13	12	ννν○○○○ννννννννννννννν○○○○○○○○○○○○ννν○ννννν	289	CAS1_DELHI	2004	NLA000400590
		ννν○○○○ννννννννννννννν○○○○○○○○○○○○νν○○ννννν	25		2005	NLA000500524
		ννννννννν○○○○○○○○○○ννννννννννννν○○○○ννννννν	149		2004	NLA000400548
14	11	ννννννννν○○○○○○○○○○ννννννννννννν○○○○νννν○νν	New	T3_ETH	2006	NLA000600430
		ννννννννν○○○○○○○○○○ννννννννννννν○○○○ννν○ννν	345		2008	NLA000800132
15	11	○○○○○○○○○○○○○○○○○○○○○○○○νννννννν○○○○ννννννν	1280	T1	2004	NLA000400046
16	11	νν○○○○○○νννννννννννν○○○○νν○○○○νν○○○○ννννννν	1607	LAM11_ZWE	2005	NLA000500458
17	10	ννν○○○○○○○○○ννννν○νννννννννννννν○○○○ννννννν	92	X3	2004	NLA000400304
		ννννννννννννννννννννννν○○○○○○○○○○○○○○○○○○○○	786		2004	NLA000401283
		νννννννννννννννννννννννννννννν○○○○○○○○○○○○○	237		2005	NLA000500740
18	10	νννννννννν○○○○ννννννν○νννννννν○○○○○○○○○○○○○	465	U	2005	NLA000500790
		νννννννννν○○○○ννν○ννν○○○○○○○○○○○○○○○○○○○○○○	New		2005	NLA000501258
		νννννννννννννννννννν○○○○○○○○○○○○○○○○○○○○○○○	402		2006	NLA000601923
		νννννννννννννννννννννννν○○○○○○○○○○○○○○○○○○○	46		2008	NLA000801472
		○○○○○○○○○○○○○○○○○○○○○○○○○○○○○○○○○○○○○○○○○○○	2669		2008	NLA000801594
		νννννννννννν○ννννννννννννννννν○ν○○○○ννννννν	36	H3-T3	2004	NLA000401512
		νννννννννννννννννννννννννννννν○ν○○○○ννννννν	50	H3	2005	NLA000500512
19	10	ννννννννννννννννννννννννν○○○○○○ν○○○○ννννννν	47	H1	2005	NLA000501842
		ννννννννννννννννννν○○ννννννννν○ν○○○○ννννννν	New	H3	2006	NLA000601244
		νννννννννννννννννν○ννννννννννν○ν○○○○ννννννν	183	H3	2006	NLA000601580
		ν○○νννννννννννννννννννννν○○○○○○ν○○○○ννννννν	1652	H1	2008	NLA000801391

24-VNTR clusters ID numbers were attributed according to their size (n°1 for the largest). Isolates ID are those stated in S1 Table. SIT = Short International Type.

All major lineages except Beijing were represented among these large clusters and no significant difference with the global representation of lineages could be detected (Fisher exact test n1 = 9, n2 = 2, p = 0.275).

### Set-up of a correspondence table between the different *M*. *tuberculosis* taxonomies and proposition of a consensual “expert” taxonomy

We took advantage of the large resulting database to examine the correspondence between the existing MTC taxonomies. Preliminarily, we removed all isolates for which no assignation existed for at least one of the taxonomy. Dataset was subsequently enriched in Afri1 and Afri2 isolates by including 52 additional isolates from a published study [80]. The resulting dataset counted 2,904 genotype profiles with classifications by TB-Lineage, Miru-Vntr*Plus*, SITVITWEB and Borile (S3 Table **)**. When browsing this material, we identified good concordance for 22 sublineages between the TB-lineage, MIRU-VNTR*Plus*, Borile and SITVITWEB classification tools. In addition, we could find a good correspondence with the SNP-based classification set up by Coll *et al*. [63] (Table 1). When assignations were discordant, we kept MIRU-VNTR*Plus* assignation only if the spoligotype signature was not contradicting it. Consequently, T5_RUS1 isolates for instance, that carry a larger deletion than standard LAM isolates in their spoligotype pattern, were classified as LAM as suggested by MIRU-VNTR*Plus*, a classification independently confirmed by Mokrousov [81]. The same rule led us to label “T2_Uganda” a part of the isolates labeled as Uganda by MIRU-VNTR*Plus*: those that carry the spacer 40-deletion in their spoligotype pattern. We named these consensual sublineages by merging short versions of the synonym assignations. For instance, isolates labeled New-1 according to MIRU-VNTR*Plus* were found mostly H4-Ural2 according to SITVITWEB, and were thus named L4_Ural2(New1) in the proposed “expert” taxonomy (Table 1). LAM3_S SITVITWEB sublineage could not find any clear correspondence in the other taxonomies; we therefore propose to temporarily abandon this sublineage and simply label the corresponding isolates “L4” as they clearly belong to the Euro-American lineage also known as lineage 4. We similarly propose to temporarily abandon MIRU-VNTR*Plus* Ghana sublineage. Our interpretation of such absence of correspondence for some sublineages with other taxonomies is that they represent an insufficient number of isolates to be relevant in a worldwide classification. Similarly, these sublineages are not described in the SNP-inspired taxonomy of Coll *et al*. [62,63].

**Fig 2:**
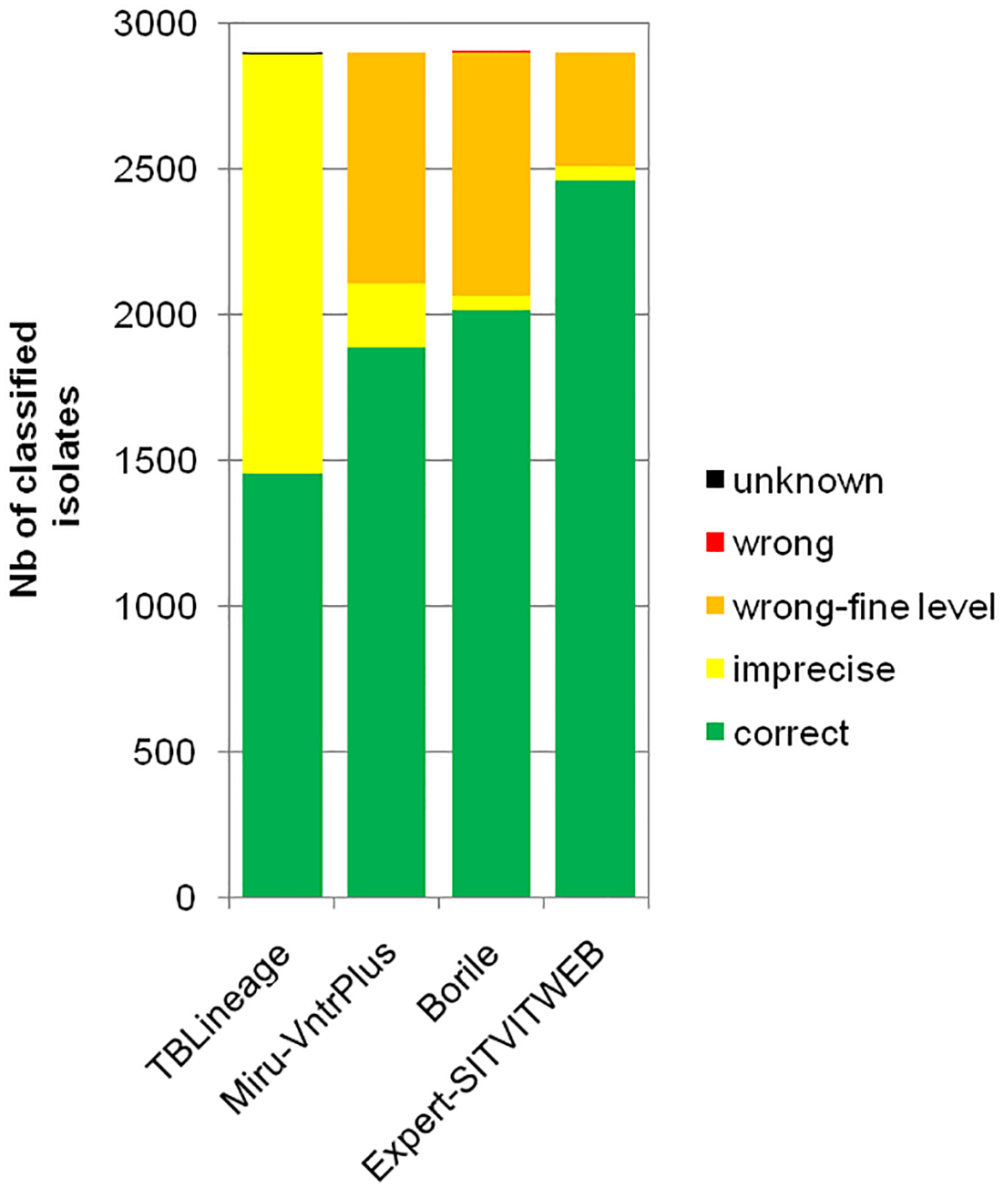
Concordance of existing classifications with the consensus classification proposed in this study.

We measured the concordance of every existing taxonomies with the newly proposed “expert” one on all isolates of our database. This consensual “expert” taxonomy reached almost perfect concordance with that of TB-lineage as retrieved using 24VNTR and spoligotype data (99.7%; discordant points n = 9; Fig 2). Although priority in naming was given to MIRU-VNTR*Plus* taxonomy during consensus building (see Methods), the “expert” taxonomy reached a very good concordance with the SITVITWEB taxonomy refined using expert knowledge (85% of concordant precise assignations; n = 2,464; Fig 2).

### Rapid machine learning algorithms to achieve fine and specific classification using spoligotyping and/or MIRU-VNTR data

Applying different classification tools on the same dataset helps understanding the real diversity in a sample, but this procedure is very time-consuming. We took advantage of our large amount of data and on machine learning to set-up a fast webtool combining all existing taxonomies including the newly proposed consensual “expert” one. We aimed at providing a classifier using spoligotype pattern, 24-VNTR profile and/or 15-MIRU profile. The 15-MIRU panel is a subset of the 24-VNTR loci with high discriminatory power. It has been validated alone or rather in combination with spoligotyping as an alternative to the 24-VNTR typing scheme for epidemiological studies [49].

We used spoligotype data to predict spoligotype-inspired taxonomies (SITVITWEB, Borile *et al*., and TB-lineage) and we used VNTR data to predict the 24-VNTR-inspired taxonomy of MIRU-VNTR*Plus*. For expert taxonomy, we chose to predict it using VNTR data as it was the first criterion inspected to set it up. Second, we reasoned that, when handling new isolates, we should check if the assignations provided by the learnt classifier obtained with Weka using the standard taxonomies are reproducing concordant associations as listed in the correspondence table set up in this study (Table 1). If they are concordant, this could comfort the expert assignation provided independently by the learnt classifier obtained with Weka. This examination of concordance between all original assignations will be thereafter referred to as “consensus classification”.

For each taxonomy, we trained five basic classifying algorithms (J48, JRip, Naïve Bayes, PART and Random Forest, see Methods), one algorithm called Vote-5 using the mode of these 5 classifications, and one algorithm called Vote-10 using the mode of these 5 classifications and their 5 meta-bagging counterparts (see Methods). The training dataset included all the 2,904 isolates described above. Accuracies of the different algorithms were stable when assessed with different levels of stratified cross-validation (S4 Table) and were impacted both by the number of lineages in the taxonomy to be inferred and by the input genotypic data (Table 3): TB-lineage with only 7 lineages was the most easy to predict, with 99.8% accuracy using the Vote-10 classifier. Spoligotype-related predictions reached higher accuracies potentially due to the simplest nature of this locus (complexity of 2^43^ as compared to more than 5^24^ *i*.*e*. a difference in complexity of more than 1,000). 15-MIRU data allowed for higher performances than 24-VNTR data when using the finest algorithms such as Vote-10, showing that phylogenetic information can be identified even among the most variable MIRU-VNTR loci.

**Table 3:** Accuracy of different induction algorithms on the training dataset using 10-fold stratified cross-validation.

Input	Predicted	Nb	Median	Induction algorithms accuracies						
data	classification	lineages/ sublineages	lineage size	J48	JRip	NB	PART	RF	Vote-5	Vote-10*
spoligo	TB-Lineage (Pred1)	7	213	99.5	99.7	98.5	99.6	99.7	99.8	99.8
	Borile (Pred3)	28	51	97.2	97.8	87.8	97.5	97.8	98.3	98.5
	SITVITWEB-expert (Pred4)	52	28	96.7	96.6	89.3	96.7	97.6	97.7	97.9
24-VNTR	MIRU-VNTR*Plus* (Pred2)	18	99	88.3	88.2	85.1	89	91.9	91	91.4
	Expert-consensus (Pred5)	24	45	86.6	80.9	80.5	87.1	90.2	88.6	88.6
15-VNTR	MIRU-VNTR*Plus* (Pred2bis)	18	99	88.2	86.1	84	88.5	91	91.8	92
	Expert-consensus (Pred5bis)	24	45	84.6	78.8	79.4	85.3	88.6	90.3	90.3

NB: Naïve Bayes. RF: Random Forest. Vote-5: Vote including the 5 algorithms shown here (from J48 to RF). Vote-10: Vote including the 5 algorithms and their meta-bagging derivatives.

*:algorithm used in Lineage Prediction tool on TBminer website. For details on the algorithm, see Material and Methods. Font size underlines performance.

On all types of genotypic data, the finest algorithm Vote-10 outperformed all basic classifiers and was therefore chosen for on-line implementation.

### Lineage Prediction available on-line on TBminer website

**Fig 3:**
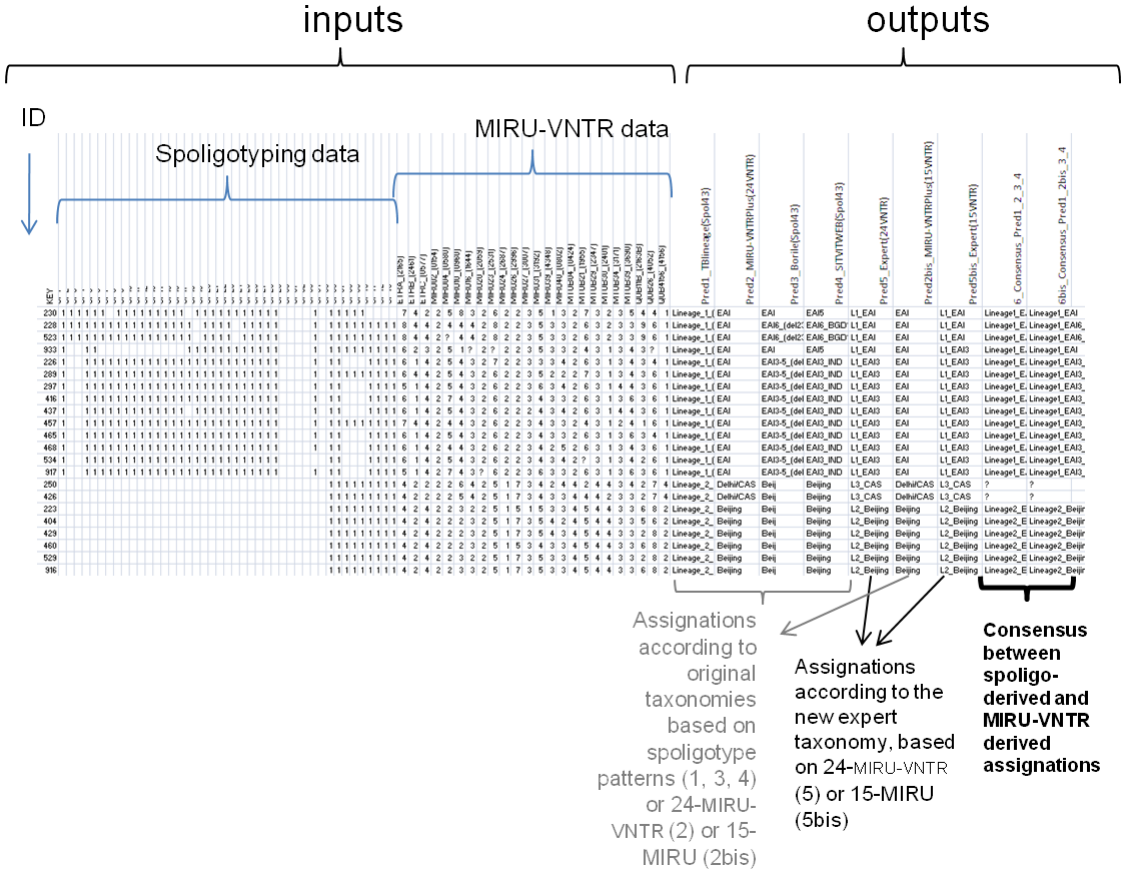
TBminer Lineage Prediction tool: the output file.

A Lineage prediction tool on the website *TBminer* was set up to make the Vote-10 classifier available to all users. Underlying rules and trees were derived using the complete training dataset. The web interface allows to upload any genotypic data including CRISPR spacer data (1 to 43 standard spacer set), and 24 standard MIRU-VNTR numbers of repeats. When including at least 43-spacers spoligotype data or 15-MIRU, TBminer Lineage Prediction webtool provides an output file with 7 new columns, one column for each of the 7 predictions mentioned in Table 3 (Pred1 to Pred5bis), as well as 2 consensus columns clarifying whether the predictions using spoligotype data and MIRU-VNTR data are concordant (Pred6 and Pred6bis; Fig 3).

### Precise performances of Lineage Prediction tool on the training set

For TB-lineage taxonomy, sensitivity and specificity of Pred1 reached 100% for all sublineages (data not shown). For MIRU-VNTR*Plus* taxonomy (Pred2 and 2bis), median sensitivity of sublineage prediction was 98.4% and 98.1% when performed on 24VNTR and 15MIRU respectively. Both predictions had very high sensitivity for all sublineages, the minimum corresponding in both cases to Ghana sublineage (90% and 85% respectively). The median specificities were 99.6% and 98.7% respectively with minima for UgandaII sublineage (93% and 90% respectively, data not shown).

For SITVITWEB-expert taxonomy that includes 50 sublineages, Pred4 predicted most sublineages with a sensitivity of 100% (n = 44) and the minimum sensitivity was 94.1% for T4_CEU1. Most sublineages also were predicted with a specificity of 100% (n = 42) and the minimum specificity was 83% for H3-T3 sublineage.

For Borile taxonomy (Pred3), sensitivity was 100% for almost all sublineages (n = 23 out of 27). Among the four sublineages that did not reach 100% sensitivity, 3 belonged to the Euro-American lineage (LAM3, LAM5-2-1(del3-13) and T3 (del13)). Twenty-five sublineages had a specificity of 100%, the minimum specificity being 95.1% for EAI3-5 (del2-3-37-38-39).

For the expert classification assessed using 24 or 15-VNTR (Pred5 and 5bis), median sensitivities were in both cases 97.6% and median specificities 98.9%. The minimum sensitivity corresponded to L4_H1-2 sublineage (83 and 78% respectively), and the minimum specificity was 83% for L4_T5 sublineage when using 24 VNTR and 80% for L1_EAI3 when using 15VNTR data. Altogether, predictions using 15VNTR data were almost as good as those using 24 loci.

### Validation of the automatic assignations on independent datasets

We tested the performance of our webtool to predict the assignations (as provided by all existing taxonomic tools and the newly proposed “expert one”) of the isolates included in the reference database of MIRU-VNTR*Plus*. This database includes not only human but also animal isolates. We first classified all isolates using the standard tools (SITVITWEB adjusted using the expert eye, MIRU-VNTR*Plus*, TB-lineage) and inferred their expert assignation as described above (Table 1). We then ran our webtool to retrieve all assignations predicted by the learnt classifier obtained with Weka.

**Fig 4:**
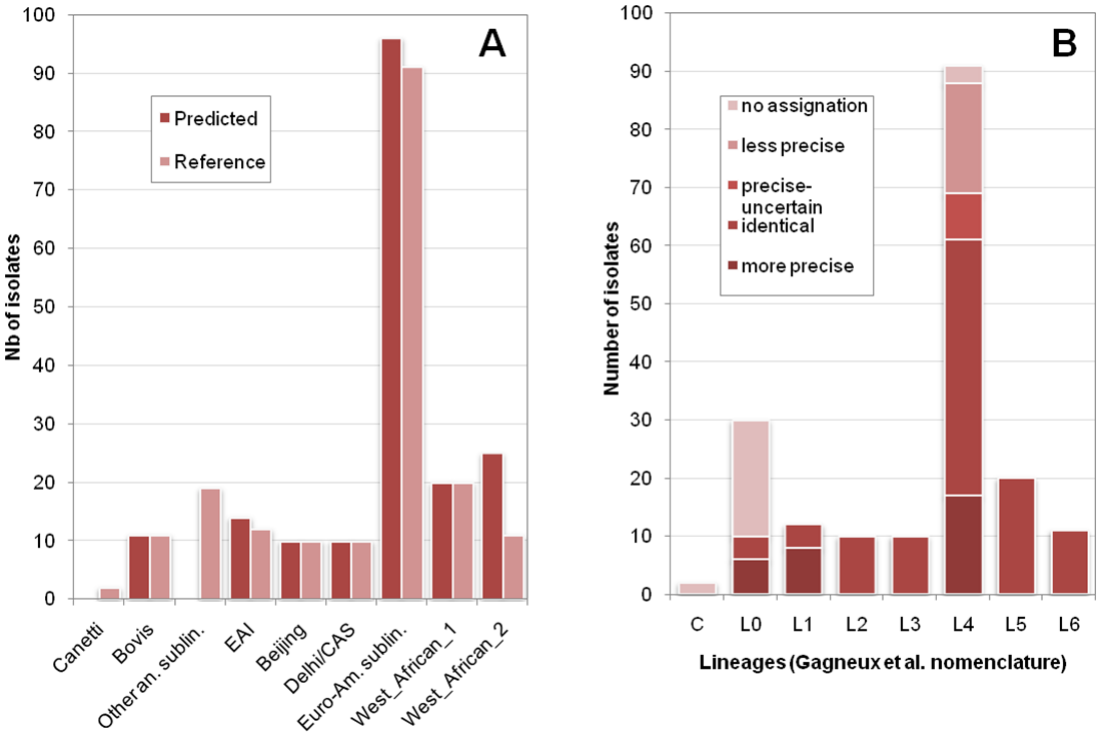
TBminer Prediction tool performance on Miru-Vntr*Plus* database. A. Concordance between TBminer Pred2_Miru-Vntr and Miru-VntrPlus assignations. B. Concordance between Pred6 and manual expert assignation accounting for original labels.

We observed that the prediction of MIRU-VNTR*Plus* assignations (Pred2 tool) had an accuracy of 100% when assigning human isolates from lineages 1 to 6 (Fig 4A). It failed in predicting animal sublineages other than *M*. *bovis* as expected due to the absence of such isolates in the training dataset. Most of them (n = 14 out of 21; 67%) were assigned to the closely related lineage Lineage6_Afri1(WestAfrican2). Altogether, diversity picture of the whole sample as provided by Pred2 tool in Lineage Prediction module of TBminer was very similar to that of MIRU-VNTR*Plus* tool, overestimating only the prevalence of West African 2 lineage and being unable to identify peculiar animal isolates as well as *M*. *canettii* (Fig 4A).

The performance of the consensus tool (consensus between spoligo and MIRU-VNTR data as appearing in column 6_Consensus_Pred1_2_3_4) was characterized by measuring its ability to reproduce the “expert” assignation. The consensus tool proved able to identify more precise labels than the expert taxonomy for most Lineage_1 isolates and a significant proportion from Lineage_0 (Bovis-BCG) and Lineage_4 (such as H1 as compared to H3) (Fig 4B). It provided a less precise assignation for less than 20% of the Lineage 4 (possibly due to a lower rate of false positive) and was unable to recognize both animal isolates other than *M*. *bovis* and *M*. *canettii* isolates. Altogether, this tool provides precise assignations for typical human isolates and is able to detect when no known/implemented lineage corresponds to the uploaded genotype.

**Fig 5:**
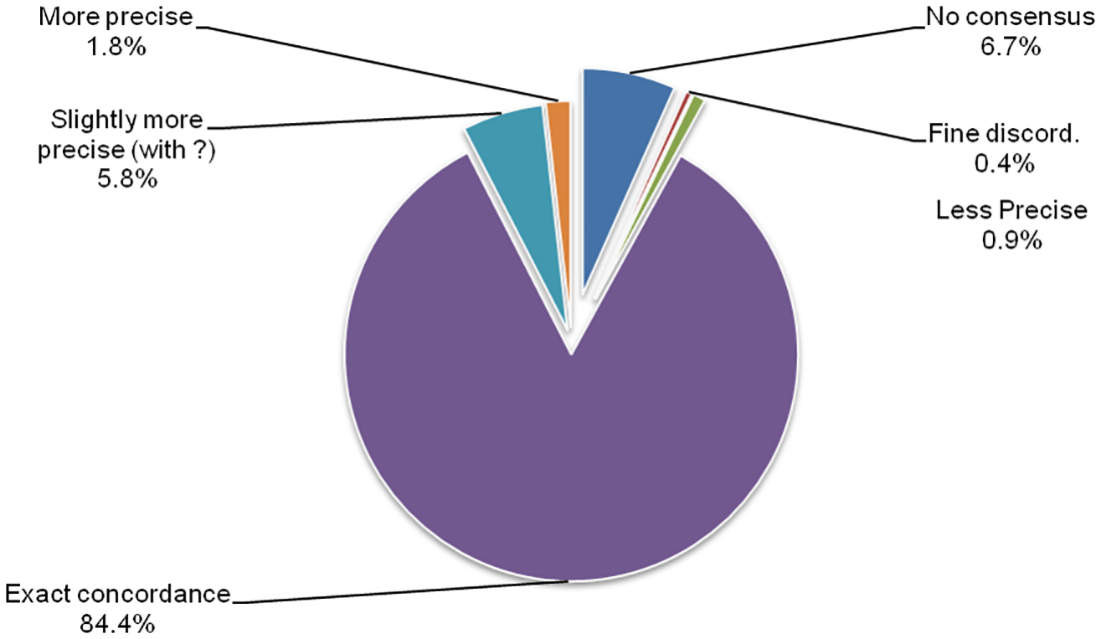
TBminer Prediction tool performance on a Pakistanis sample. Consensus Lineage Prediction tool of TBminer was compared to the Expert assignation on an independent dataset from Pakistan.

We performed the same approach on a representative dataset of Pakistanis clinical isolates for which complete genotypes (at most one VNTR lacking) were available (n = 225). This dataset is interesting because it uncovers human isolates of an origin very different from that of the dataset used to set up TBminer Lineage Prediction webtool. Our rationale was that, if good performances could be observed with this dataset, this would mean that TBminer Lineage Prediction tool is robust to the geographical origin of the isolates. For SITVITWEB taxonomy, Pred4 reached an accuracy of 99.5% (1 error out of 190 isolates, mispredicting a T2 assignation for a LAM3 genotype). For isolates with no prototypic assignation, examination of the profiles found the predicted assignation to be plausible for 83% of the isolates (n = 29 out of 35), so that altogether, 28 isolates (12.4%) reached a better assignation using TBminer than using SITVITWEB. For MIRU-VNTR*Plus* taxonomy, Lineage prediction tool Pred2 provided a concordance of 94.1% with the reference assignation using 24-VNTR genotypes, and reached 99.4% concordance for the most prevalent lineage (Delhi/CAS). Concordance was low for isolates exhibiting spoligotype patterns apparently discordant with their MIRU-VNTR profiles: for instance, isolates classified as Cameroon by MIRU-VNTR*Plus* lacked the deletion of spacers 23 to 25 in their spoligotype pattern, the deletion that originally characterizes this sublineage [82,83]. This suggests that no appropriate label exists in the current system for the corresponding isolates. In this case, the consensus proposed by TBminer, “Lineage4”, may be more accurate than the tentative discordant assignations proposed by the existing taxonomies. The Consensus tool (6_Consensus) exhibited a perfect concordance with the expert assignation for 92% of the isolates, 6% of which being proposed a finer assignation by the automatized tool only (Fig 5). Potential errors of this consensus tool were very rare and limited to sublineage inside Lineage 4 (1.3%). For 6.7% of the isolates, no output could be provided by this tool: no consensus was identified between MIRU-VNTR-based and spoligotype pattern-based classifications. These isolates need further studies, either using SNP or using expert knowledge of the genetic diversity in the region. For instance, we identified isolates carrying a Beijing spoligotype but a VNTR profile characteristic of CAS isolates. As recent studies have identified Pseudo-Beijing isolates in the Middle East region, these specific isolates might well represent new cases of this sublineage identified as a bona-fide Lineage3-CAS [84]. Interestingly, the consensus based on 15-MIRU and spoligotype (6bis) performed almost as well as that using 24-VNTR (91.6% instead of 92% concordance), with the only discordance reported for 24-VNTR being moved to the unassigned group (No consensus) (data not shown).

We proceeded to a third validation test using the SNP-classification provided by Abadia *et al*. [56]. In that study, several SIT were shown to be associated to phylogenetic SNPs classifying them differently than SITVITWEB. For instance, SIT316 previously labeled as Haarlem 1 because of lacking spacers 26–31 (but also lacking spacer 25 and spacer 40) was found to be related to the T2 sublineage (absence of the *mgtC* SNP characteristic for Haarlem sublineage and presence of the *recR* specific to T2). Similarly, SIT254, originally classified as T5-RUS1 was renamed LAM because of carrying the *ligB* mutation. This SIT harbors the very specific signature of LAM (21–24 spacers deletion) but it was not recognized as such until 2014 because of other missing spacers [56,81]. Here, we compared the SITVITWEB assignations, the expert assignation taking into account new knowledge such as the belonging of former T5_RUS1 group to LAM sublineage [81], the SNP naming, and the Consensus assignation provided by Lineage Prediction (6 and 6bis) for isolates having a discordant naming according to SITVITWEB and SNP taxonomies as found in [56]. We also included 6 isolates harbouring SIT742 as they are likely highly related to SIT316, and therefore likely belonging to the same SNP lineage. Out of 21 isolates, 12 had a match between the SNP naming and the Lineage Prediction (6) naming (57%, see Table 4). Two isolates only (10%), annotated as Haarlem 3 under SITVITWEB taxonomy and classified as H by our algorithm were classified as X by SNP classification, the other being linked to an imprecise but not spurious assignation (Table 4).

**Table 4:** Concordance between SNP classification and the newly proposed and automatized consensus tool on a set of isolates with conflicting assignations in existing taxonomies.

		Lineage or Sublineage			Concor-dance	
SIT	Spoligotype pattern	classical (SITVIT-WEB)	SNP (Abadia *et al*.)	TBminer Consensus Lineage Prediction	between SNP and Consensus Lineage Pred	N
254	νννννννννννννν○○○○○○○○○○νννννννν○○○○ννννννν	T5_RUS1	LAM	Lineage4_LAM	**+++**	9
316	νννννννννννννννννννννννν○○○○○○○ν○○○○ννν○ννν	H3	T2	Lineage4_New1 (Ural2)?	**+/-**	2
316	νννννννννννννννννννννννν○○○○○○○ν○○○○ννν○ννν	H3	T2	Lineage4-T2?_H?	**+**	4
1531	ννννννννννννννννν○νννννννννννννν○○○○○○○νννν	U	X	Lineage4_X	**+++**	3
134	νννννννννννννννννννννννννννννν○ν○○○○νν○○ννν	H3	X	Lineage4_H3	-	2
78	νννννννννννννννννννννννννννννννν○○○○ννν○○νν	T1-T2	Tur	Lineage4	**+/-**	1
1274	ννννννννννννννννννννννν○○○○○○○○○○○○○○○○νννν	U	H	Lineage4_H?	**+**	1

The only available genotypic data available to perform classification was spoligotype patterns.

## Discussion

We characterized *M*. *tuberculosis* complex diversity in the Netherlands using the 3 most widely-used genotyping techniques, 24-MIRU-VNTR and spoligotyping and IS*611*0-RFLP on a set of clinical isolates collected over 5 years (2004–2008). To analyze this diversity we set up an automatic Cluster analysis tool available on a new website dedicated to molecular epidemiology and classification for human tuberculosis, called TBminer. Combined with a few additional genotypes from Lineages 5 and 6 (*M*. *africanum* lineages), the resulting database allowed us to identify correspondences between the existing taxonomies based on spoligotype or MIRU-VNTR data, leading to the setting up of a consensual “expert” taxonomy. We then set up a tool based on machine learning for identifying the alternative assignations of new isolates according to these different taxonomies. This tool is available as “Lineage Prediction” on the TBminer website.

### *M*. *tuberculosis* complex diversity in the Netherlands

Our study confirmed the large prevalence of the Euro-American lineage in the Netherlands as observed in Western countries and Africa, and especially so called “T” isolates [25]. Beijing prevalence proved stable as compared to the 1993–2000 period, accounting for 6.6% of the TB cases as compared to between 5 and 8% depending on the year between 1995 and 2000 [85]. Although tuberculosis prevalence steadily decreases among inlanders and increases among immigrants i.e. *M*. *tuberculosis* strains are more and more collected from non-Western European citizens [86], this features had no significant consequence on lineage prevalence by now. Recent transmission rate differed if evaluated on a one-year period or over a five year period. This reminds that the concept of recent transmission should be used cautiously because current inland transmission may reveal only few years after having occurred [77]. Alternatively, this observation might be due to active transmission in the original country of immigrants.

### Consensus taxonomy for *M*. *tuberculosis* complex

Subclassification of *M*. *tuberculosis* complex has developed thanks to the use of high-mutation rate markers: IS*6110*-RFLP, CRISPR, MIRU-VNTR. The monophyly of groups defined using spoligotype patterns (CRISPR diversity) or multiple MIRU-VNTR genotyping was questioned due to the possibility of convergence in these loci. Some sublineages such as LAM were in fact wrongly delimited as evidenced by discordant genotypes using the alternative genotyping method: LAM10_CAM isolates grouped with other LAM according to spoligotyping carry MIRU-VNTR patterns largely discordant with other LAMs, and T5_RUS1 isolates clearly distinguished from LAM according to spoligotyping carried in contrasts patterns that indicated a clear relatedness with LAM [81]. The redefinitions of groups were confirmed by the use of SNPs [62,63]. SNP subclassification tends thus to become the new standard. As its cost when using complete SNP panels is still high, a combination of a global panel and a restricted panel using geographically-specific SNPs could become future practice for correct and precise taxonomical assignation of any local TB clinical isolate.

Confronting VNTR markers with assignation based on spoligotype patterns can still provide helpful information for classifying new isolates. The large database set up in this study allowed us to build a new classification tool efficient when used on both 15-MIRU-VNTR and spoligotype patterns. This on-line induction algorithm proved fairly robust. We believe that until Whole Genome Sequencing decreases to below 40 euros per isolate, the continuous use of 15-MIRU-VNTR typing in combination with spoligotyping is relevant to both infer main epidemiological events and get a clear picture of the circulating diversity. As our tool greatly speeds up the bioinformatical analysis of produced genotyping patterns, it may be of great use to epidemiologists.

A limitation to our tool is that it was built exclusively on widespread human *M*. *tuberculosis* lineages, excluding animal sublineages such as *M*. *caprae*, *M*. *microtii*, *M*. *pinnipedii*, as well as *M*. *canettii* and the recently described Lineage7 isolates [16]. *M*. *caprae* and *M*. *microtii* genotypes from MIRU-VNTR*Plus* database were confidently classified as Lineage 0 or Lineage 6 by our tool. Most *M*. *canettii* genotypes (21-VNTR data) as available in Blouin *et al*. [18] found no concordant assignation by Pred2, Pred2bis, Pred5 and Pred5bis in our Lineage prediction tool (n = 60; 75%), 15% were erroneously assigned to L4 Lineage, 4% to EAI_Lineage 1 and 1% to Animal lineage_Lineage 0 (data not shown). Altogether, we can infer that our Lineage Prediction tool as available on TBminer website is 100% reliable for large lineage assignation based on 15-MIRU and spoligotyping when implemented on human epidemiological datasets in most regions of the world. The only possible region where our tool may provide little information and likely some spurious information could be the Horn of Africa where the diversity deviates from the data we used to implement our tool.

### Search for consensus taxonomies among Bacteria using Machine Learning

**Fig 6:**
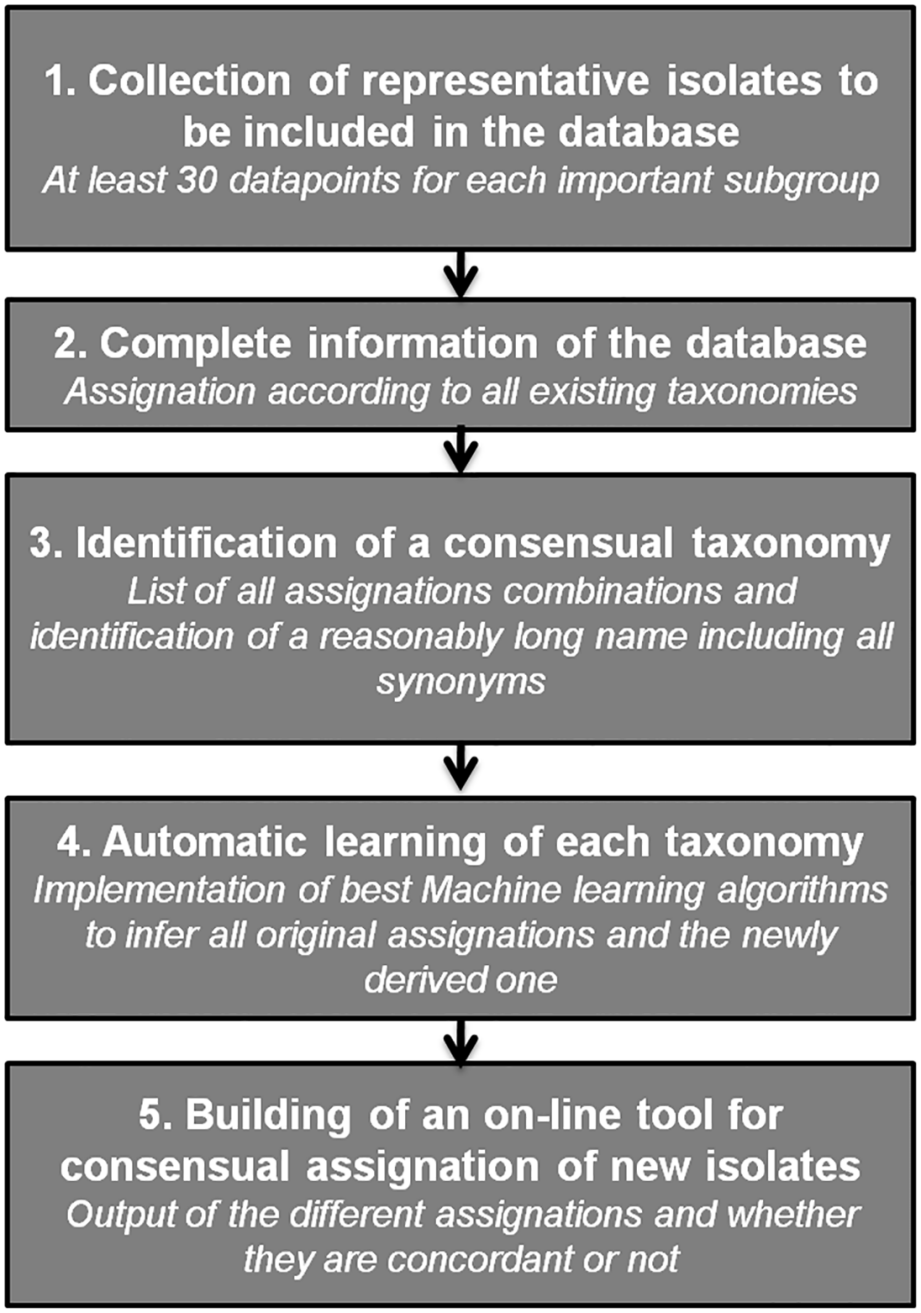
Approach for consensus building between conflictive taxonomies.

Taxonomies rely on the biological data characterizing the classified organisms. When new tools are developed to characterize diversity, new taxonomies are usually set up. It is only when confronting multiple information that a consensus can emerge. Taxonomies built on different genetic data do not match either due to convergence events or due to horizontal gene transfer. Dealing with a single taxonomy without trying to search for a consensus limits inferences on the properties of lineages as some characteristics may have been attributed to a complete subgroup defined by taxonomy A, but a subpart that classification B could have identified may really carry the property of interest. Building a consensus is usually a long process. We think that our approach can speed up definition of consensual taxonomies. We recommend the following steps as developed in this study (Fig 6): 1) set up of a large collection of isolates representative of the largest diversity and type them with all available tools; 2) inform every alternative assignation according to existing taxonomies; 3) identify the concordant assignations, and propose a name made of all synonyms (but of reasonable length) for the consensual groups; 4) implement Machine learning algorithms, for instance in Weka, to learn all taxonomies independently; 5) Making the tool for consensus assignation available on-line. This last step of making the consensus available on-line, is in our opinion, very important as it will clearly help all users to get acquainted with it. By providing not only the consensus assignation but also the assignations according to existing taxonomies, users can build their trust in the consensus by checking if the assignation provided for the known taxonomy is relevant to their expert knowledge.

Given the deluge of genomic data produced using next generation sequencing, our approach could also be used to check SNPs informativity in a very near future. Indeed, SNPs diversity may cover various level of informativity, which remains for the time-being poorly explored at the statistical level, in particular concerning epistatic mutations significance.

## Conclusion

We developed an approach making use of standard typing data for human *M*. *tuberculosis* isolates to infer a consensual taxonomy concordant with most up-to-date data on Whole Genome Sequencing diversity. We believe that this tool will not only increase the understanding of clinical and epidemiological experts about the tuberculosis worldwide diversity, but it will also help them build refined knowledge on the genetic diversity circulating in their country. We hope that the same approach can benefit other human pathogens having alternative taxonomies according to Serotypes, CRISPRtypes, MLST-types such as *Salmonella*, *Listeria*, *Brucella*, and more broadly to other organisms such as bacterial plant-pathogens.

## Supporting Information

S1 Fig Comparative discriminatory power of the standard genotyping techniques when used in combination. (DOCX)

S1 Table Genotypes and assignations of the 3,454 tuberculous isolates collected and cultured by the Netherlands National Reference center for tuberculosis between 2004 and 2008. (XLS)

S2 Table Clustering rate in the Netherlands from 2004 to 2008, as assessed using different genotyping techniques, per year and globally. (DOCX)

S3 Table Genotypes and assignations of the 2,904 isolates used to set up TBminer prediction tool. (XLSX)

S4 Table Accuracy of Lineage prediction tools as a function of the number of folds during stratified cross-validation. (XLSX)

## References

[1] Wayne LG (1984) Mycobacterial speciation In: Wayne GPKaLG, editor. The mycobacteria: asourcebook, Par A. New York: Marcel Dekker Inc pp. 25–65.

[2] Stackebrandt E, Goebel BM (1997) Taxonomic note: a place of DNA-DNA reassociation and 16S rRNA sequence analysis in the present species definition in bacteriology. Int J Syst Bact 44: 846–849.

[3] Stackebrandt E, Goebel BM (1994) Taxonomic Note: A Place for DNA-DNA Reassociation and 16s rRNA Sequence Analysis in the Present Species Definition in Bacteriology. Int J Syst Bact 44: 846–849.

[4] Goris J, Konstantinidis KT, Klappenbach JA, Coenye T, Vandamme P, Tiedje JM (2007) DNA-DNA hybridization values and their relationship to whole-genome sequence similarities. Int J Syst Evol Microbiol 57: 81–91. 17220447 10.1099/ijs.0.64483-0

[5] Lehmann K, R N (1907) Lehmann's Medizin Handatlanten X. Atlas and Grundriss der Bakteriologie une Lehrbuch der speciellen backteriologischen Diagnostik; Lehmann JF, editor. Munich.

[6] Reed G (1957) Genus Mycobacterium (species affecting warm-blooded animals except those causing leprosy In: Breed RSM, E.G.D., Smith N.R., editor. Bergey's Manual of Determinative Bacteriology. 7th ed ed. Baltimore: Williams and Wilkins pp. 703–704.

[7] Downey J (1962) Host-Plant relations as data for butterfly classification. Systematic Zoology 11: 150–159.

[8] Karlson A, Lessel E (1970) Mycobacterium bovis Nom. Nov. Int J Syst Bacteriology 20: 273–282.

[9] Aranaz A, Liebana E, Gomez-Mampaso E, Galan JC, Cousins D, Ortega A, et al (1999) *Mycobacterium tuberculosis* subsp. caprae subsp. nov.: a taxonomic study of a new member of the *Mycobacterium tuberculosis* complex isolated from goats in Spain. Int J Syst Bacteriol 49: 1263–1273. 10425790 10.1099/00207713-49-3-1263

[10] Cousins DV, Bastida R, Cataldi A, Quse V, Redrobe S, Dow S, et al (2003) Tuberculosis in seals caused by a novel member of the Mycobacterium tuberculosis complex: Mycobacterium pinnipedii sp. nov. Int J Syst Evol Microbiol 53: 1305–1314. 13130011 10.1099/ijs.0.02401-0

[11] Alexander KA, Laver PN, Michel AL, Williams M, van Helden PD, Warren RM, et al (2010) Novel Mycobacterium tuberculosis complex pathogen, M. mungi. Emerg Infect Dis 16: 1296–1299. 10.3201/eid1608.100314 20678329 PMC3298296

[12] van Ingen J, Rahim Z, Mulder A, Boeree MJ, Simeone R, Brosch R, et al (2012) Characterization of Mycobacterium orygis as M. tuberculosis complex subspecies. Emerg Infect Dis 18: 653–655. 10.3201/eid1804.110888 22469053 PMC3309669

[13] Parsons SD, Drewe JA, Gey van Pittius NC, Warren RM, van Helden PD (2013) Novel cause of tuberculosis in meerkats, South Africa. Emerg Infect Dis 19: 2004–2007. 10.3201/eid1912.130268 24274183 PMC3840885

[14] Castets M, Boisvert H, Grumbach F, Brunel M, Rist N (1968) Les bacilles tuberculeux de type africain: note préliminaire. Rev Tuberc Pneumol 32: 179–184. 4985104

[15] van Soolingen D, Hoogenboezem T, de Haas PE, Hermans PW, Koedam MA, Teppema KS, et al (1997) A novel pathogenic taxon of the Mycobacterium tuberculosis complex, Canetti: characterization of an exceptional isolate from Africa. Int J Syst Bacteriol 47: 1236–1245. 9336935 10.1099/00207713-47-4-1236

[16] Blouin Y, Hauck Y, Soler C, Fabre M, Vong R, Dehan C, et al (2012) Significance of the identification in the Horn of Africa of an exceptionally deep branching Mycobacterium tuberculosis clade. PLoS One 7: e52841 10.1371/journal.pone.0052841 23300794 PMC3531362

[17] Supply P, Marceau M, Mangenot S, Roche D, Rouanet C, Khanna V, et al (2013) Genomic analysis of smooth tubercle bacilli provides insights into ancestry and pathoadaptation of Mycobacterium tuberculosis. Nat Genet 45: 172–179. 10.1038/ng.2517 23291586 PMC3856870

[18] Blouin Y, Cazajous G, Dehan C, Soler C, Vong R, Hassan MO, et al (2014) Progenitor "Mycobacterium canettii" clone responsible for lymph node tuberculosis epidemic, Djibouti. Emerg Infect Dis 20: 21–28. 10.3201/eid2001.130652 24520560 PMC3884719

[19] Laszlo A, Gill P, Handzel V, Hodgkin MM, Helbecque DM (1983) Conventional and radiometric drug susceptibility testing of Mycobacterium tuberculosis complex. J Clin Microbiol 18: 1335–1339. 6418755 10.1128/jcm.18.6.1335-1339.1983 PMC272903

[20] Wirth T, Hildebrand F, Allix-Beguec C, Wolbeling F, Kubica T, Kremer K, et al (2008) Origin, spread and demography of the Mycobacterium tuberculosis complex. PLoS Pathog 4: e1000160 10.1371/journal.ppat.1000160 18802459 PMC2528947

[21] van Embden JD, Cave MD, Crawford JT, Dale JW, Eisenach KD, Gicquel B, et al (1993) Strain identification of Mycobacterium tuberculosis by DNA fingerprinting: recommendations for a standardized methodology. J Clin Microbiol 31: 406–409. 8381814 10.1128/jcm.31.2.406-409.1993 PMC262774

[22] Groenen PM, Bunschoten AE, van Soolingen D, van Embden JD (1993) Nature of DNA polymorphism in the direct repeat cluster of Mycobacterium tuberculosis; application for strain differentiation by a novel typing method. Mol Microbiol 10: 1057–1065. 7934856 10.1111/j.1365-2958.1993.tb00976.x

[23] Kamerbeek J, Schouls L, Kolk A, van Agterveld M, van Soolingen D, Kuijper S, et al (1997) Simultaneous detection and strain differentiation of Mycobacterium tuberculosis for diagnosis and epidemiology. J Clin Microbiol 35: 907–914. 9157152 10.1128/jcm.35.4.907-914.1997 PMC229700

[24] Sola C, Devallois A, Horgen L, Maisetti J, Filliol I, Legrand E, et al (1999) Tuberculosis in the Caribbean: using spacer oligonucleotide typing to understand strain origin and transmission. Emerg Infect Dis 5: 404–414. 10341177 10.3201/eid0503.990311 PMC2640778

[25] Brudey K, Driscoll J, Rigouts L, Prodinger WM, Gori A, Al Hajoj SA, et al (2006) Mycobacterium tuberculosis complex genetic diversity: mining the fourth international spoligotyping database (SpolDB4) for classification, Population Genetics, and Epidemiology. BMC Microbiol 6: 23 16519816 10.1186/1471-2180-6-23 PMC1468417

[26] Demay C, Liens B, Burguière T, Hill V, Couvin D, Millet J, et al (2012) SITVITWEB—A publicly available international multimarker database for studying Mycobacterium tuberculosis genetic diversity and molecular epidemiology. Infect Genet Evol in Press. 10.1016/j.meegid.2012.02.004 22365971

[27] van Embden JDA, van Gorkom T, Kremer K, Jansen R, van der Zeijst BAM, Schouls LM, et al (2000) Genetic variation and evolutionary origin of the Direct repeat locus of *Mycobacterium tuberculosis* complex bacteria. J Bacteriol 182: 2393–2401. 10762237 10.1128/jb.182.9.2393-2401.2000 PMC111299

[28] van der Zanden AG, Kremer K, Schouls LM, Caimi K, Cataldi A, Hulleman A, et al (2002) Improvement of differentiation and interpretability of spoligotyping for Mycobacterium tuberculosis complex isolates by introduction of new spacer oligonucleotides. J Clin Microbiol 40: 4628–4639. 12454164 10.1128/JCM.40.12.4628-4639.2002 PMC154657

[29] Reyes JF, Francis AR, Tanaka MM (2008) Models of deletion for visualizing bacterial variation: an application to tuberculosis spoligotypes. BMC Bioinformatics 9: 496 10.1186/1471-2105-9-496 19036166 PMC2620273

[30] Streicher EM, Victor TC, van der Spuy G, Sola C, Rastogi N, van Helden PD, et al (2007) Spoligotype signatures in the Mycobacterium tuberculosis complex. J Clin Microbiol 45: 237–240. 17065260 10.1128/JCM.01429-06 PMC1828946

[31] van Soolingen D, Qian L, de Haas PE, Douglas JT, Traore H, Portaels F, et al (1995) Predominance of a single genotype of Mycobacterium tuberculosis in countries of east Asia. J Clin Microbiol 33: 3234–3238. 8586708 10.1128/jcm.33.12.3234-3238.1995 PMC228679

[32] Kremer K, van Soolingen D, Frothingham R, Haas WH, Hermans PW, Martin C, et al (1999) Comparison of methods based on different molecular epidemiological markers for typing of Mycobacterium tuberculosis complex strains: interlaboratory study of discriminatory power and reproducibility. J Clin Microbiol 37: 2607–2618. 10405410 10.1128/jcm.37.8.2607-2618.1999 PMC85295

[33] Warren RM, Streicher EM, Sampson SL, Van Der Spuy GD, Richardson M, Nguyen D, et al (2002) Microevolution of the Direct Repeat Region of Mycobacterium tuberculosis: Implications for Interpretation of Spoligotyping Data. J Clin Microbiol 40: 4457–4465. 12454136 10.1128/JCM.40.12.4457-4465.2002 PMC154636

[34] Comas I, Homolka S, Niemann S, Gagneux S (2009) Genotyping of genetically monomorphic bacteria: DNA sequencing in mycobacterium tuberculosis highlights the limitations of current methodologies. PLoS One 4: e7815 10.1371/journal.pone.0007815 19915672 PMC2772813

[35] Kato-Maeda M, Gagneux S, Flores LL, Kim EY, Small PM, Desmond EP, et al (2011) Strain classification of Mycobacterium tuberculosis: congruence between large sequence polymorphisms and spoligotypes. Int J Tuberc Lung Dis 15: 131–133. 21276309 PMC3600895

[36] Borile C, Labarre M, Franz S, Sola C, Refregier G (2011) Using affinity propagation for identifying subspecies among clonal organisms: lessons from M. tuberculosis. BMC Bioinformatics 12: 224 10.1186/1471-2105-12-224 21635750 PMC3126747

[37] Vitol I, Driscoll J, Kreiswirth B, Kurepina N, Bennett KP (2006) Identifying Mycobacterium tuberculosis complex strain families using spoligotypes. Infect Genet Evol 6: 491–504. 16632413 10.1016/j.meegid.2006.03.003

[38] Shabbeer A, Cowan LS, Ozcaglar C, Rastogi N, Vandenberg SL, Yener B, et al (2012) TB-Lineage: an online tool for classification and analysis of strains of Mycobacterium tuberculosis complex. Infect Genet Evol 12: 789–797. 10.1016/j.meegid.2012.02.010 22406225

[39] Hershberg R, Lipatov M, Small PM, Sheffer H, Niemann S, Homolka S, et al (2008) High functional diversity in Mycobacterium tuberculosis driven by genetic drift and human demography. PLoS Biol 6: e311 10.1371/journal.pbio.0060311 19090620 PMC2602723

[40] Brosch R, Gordon SV, Marmiesse M, Brodin P, Buchrieser C, Eiglmeier K, et al (2002) A new evolutionary scenario for the Mycobacterium tuberculosis complex. Proc Natl Acad Sci U S A 99: 3684–3689. 11891304 10.1073/pnas.052548299 PMC122584

[41] Tsolaki AG, Hirsh AE, DeRiemer K, Enciso JA, Wong MZ, van Soolingen D, et al (2004) Functional and evolutionary genomics of Mycobacterium tuberculosis: Insights from genomic deletions in 100 strains. Proc Natl Acad Sci U S A 101: 4865–4870. Epub 2004 Mar 4815. 15024109 10.1073/pnas.0305634101 PMC387340

[42] Frothingham R, Meeker-O'Connell WA (1998) Genetic diversity in the Mycobacterium tuberculosis complex based on variable numbers of tandem DNA repeats. Microbiology 144 (Pt 5): 1189–1196. 9611793 10.1099/00221287-144-5-1189

[43] Supply P, Mazars E, Lesjean S, Vincent V, Gicquel B, Locht C. (2000) Variable human minisatellite-like regions in the *Mycobacterium tuberculosis* genome. Mol Microbiol 36: 762–771. 10844663 10.1046/j.1365-2958.2000.01905.x

[44] Mazars E, Lesjean S, Banuls AL, Gilbert M, Vincent V, Gicquel B, et al (2001) High-resolution minisatellite-based typing as a portable approach to global analysis of Mycobacterium tuberculosis molecular epidemiology. Proc Natl Acad Sci U S A 98: 1901–1906. 11172048 10.1073/pnas.98.4.1901 PMC29354

[45] Sola C, Filliol I, Legrand E, Lesjean S, Locht C, Supply P, et al (2003) Genotyping of the Mycobacterium tuberculosis complex using MIRUs: association with VNTR and spoligotyping for molecular epidemiology and evolutionary genetics. Infect Genet Evol 3: 125–133. 12809807 10.1016/s1567-1348(03)00011-x

[46] Allix-Beguec C, Fauville-Dufaux M, Supply P (2008) Three-year population-based evaluation of standardized mycobacterial interspersed repetitive-unit-variable-number tandem-repeat typing of Mycobacterium tuberculosis. J Clin Microbiol 46: 1398–1406. 10.1128/JCM.02089-07 18234864 PMC2292969

[47] Weniger T, Krawczyk J, Supply P, Niemann S, Harmsen D (2010) MIRU-VNTRplus: a web tool for polyphasic genotyping of Mycobacterium tuberculosis complex bacteria. Nucleic Acids Res 38: W326–331. 10.1093/nar/gkq351 20457747 PMC2896200

[48] Schurch AC, Kremer K, Kiers A, Boeree MJ, Siezen RJ, van Soolingen D. (2011) Preferential deletion events in the direct repeat locus of Mycobacterium tuberculosis. J Clin Microbiol 49: 1318–1322. 10.1128/JCM.01848-10 21325559 PMC3122804

[49] Supply P, Allix C, Lesjean S, Cardoso-Oelemann M, Rusch-Gerdes S, Willery E, et al (2006) Proposal for Standardization of Optimized Mycobacterial Interspersed Repetitive Unit-Variable-Number Tandem Repeat Typing of Mycobacterium tuberculosis. J Clin Microbiol 44: 4498–4510. 17005759 10.1128/JCM.01392-06 PMC1698431

[50] Allix-Beguec C, Harmsen D, Weniger T, Supply P, Niemann S (2008) Evaluation and user-strategy of MIRU-VNTRplus, a multifunctional database for on-line analysis of genotyping data and phylogenetic identification of Mycobacterium tuberculosis complex isolates. J Clin Microbiol. 10.1128/JCM.00540-08 PMC2519508 18550737

[51] Kremer K, Arnold C, Cataldi A, Gutierrez MC, Haas WH, Panaiotov S, et al (2005) Discriminatory power and reproducibility of novel DNA typing methods for Mycobacterium tuberculosis complex strains. J Clin Microbiol 43: 5628–5638. 16272496 10.1128/JCM.43.11.5628-5638.2005 PMC1287774

[52] Cardoso Oelemann M, Gomes HM, Willery E, Possuelo L, Batista Lima KV, Allix-Beguec, et al (2011) The forest behind the tree: phylogenetic exploration of a dominant Mycobacterium tuberculosis strain lineage from a high tuberculosis burden country. PLoS One 6: e18256 10.1371/journal.pone.0018256 21464915 PMC3064675

[53] Yasmin M, Gomgnimbou MK, Siddiqui RT, Refregier G, Sola C (2014) Multi-drug resistant Mycobacterium tuberculosis complex genetic diversity and clues on recent transmission in Punjab, Pakistan. Infection Genetics and Evolution 27: 6–14. 10.1016/j.meegid.2014.06.017 24981519

[54] Evans JT, Serafino Wani RL, Anderson L, Gibson AL, Smith EG, Wood A, et al (2011) A geographically-restricted but prevalent Mycobacterium tuberculosis strain identified in the West Midlands Region of the UK between 1995 and 2008. PLoS One 6: e17930 10.1371/journal.pone.0017930 21464965 PMC3064665

[55] Filliol I, Driscoll JR, van Soolingen D, Kreiswirth BN, Kremer K, Valetudie G, et al (2003) Snapshot of moving and expanding clones of Mycobacterium tuberculosis and their global distribution assessed by spoligotyping in an international study. J Clin Microbiol 41: 1963–1970. 12734235 10.1128/JCM.41.5.1963-1970.2003 PMC154710

[56] Abadia E, Zhang J, Vultos TD, Ritacco V, Kremer K, Aktas E, et al (2010) Resolving lineage assignation on Mycobacterium tuberculosis clinical isolates classified by spoligotyping with a new high-throughput 3R SNPs based method. Infect Genet Evol 10 1066–1074. 10.1016/j.meegid.2010.07.006 20624486

[57] Homolka S, Projahn M, Feuerriegel S, Ubben T, Diel R, Dafae F, et al (2012) High resolution discrimination of clinical Mycobacterium tuberculosis complex strains based on single nucleotide polymorphisms. PLoS One 7: e39855 10.1371/journal.pone.0039855 22768315 PMC3388094

[58] Niemann S, Koser CU, Gagneux S, Plinke C, Homolka S, Bignell H, et al (2009) Genomic diversity among drug sensitive and multidrug resistant isolates of Mycobacterium tuberculosis with identical DNA fingerprints. PLoS One 4: e7407 10.1371/journal.pone.0007407 19823582 PMC2756628

[59] Walker TM, Ip CL, Harrell RH, Evans JT, Kapatai G, Dedicoat MJ, et al (2013) Whole-genome sequencing to delineate Mycobacterium tuberculosis outbreaks: a retrospective observational study. Lancet Infect Dis 13: 137–146. 10.1016/S1473-3099(12)70277-3 23158499 PMC3556524

[60] Bryant JM, Schurch AC, van Deutekom H, Harris SR, de Beer JL, de Jager V, et al (2013) Inferring patient to patient transmission of Mycobacterium tuberculosis from whole genome sequencing data. BMC Infect Dis 13: 110 10.1186/1471-2334-13-110 23446317 PMC3599118

[61] Schurch AC, Kremer K, Daviena O, Kiers A, Boeree MJ, Siezen RJ, et al (2010) High resolution typing by integration of genome sequencing data in a large tuberculosis cluster. J Clin Microbiol 48: 3403–3406. 10.1128/JCM.00370-10 20592143 PMC2937716

[62] Coll F, McNerney R, Guerra-Assuncao JA, Glynn JR, Perdigao J, Viveiros M, et al (2014) A robust SNP barcode for typing Mycobacterium tuberculosis complex strains. Nat Commun 5: 4812 10.1038/ncomms5812 25176035 PMC4166679

[63] Coll F, Preston M, Guerra-Assuncao JA, Hill-Cawthorn G, Harris D, Perdigao J, et al (2014) PolyTB: a genomic variation map for Mycobacterium tuberculosis. Tuberculosis (Edinb) 94: 346–354. 24637013 10.1016/j.tube.2014.02.005 PMC4066953

[64] Amancio DR, Comin CH, Casanova D, Travieso G, Bruno OM, Rodrigues FA, et al (2014) A systematic comparison of supervised classifiers. PLoS One 9: e94137 10.1371/journal.pone.0094137 24763312 PMC3998948

[65] Sebban M, Mokrousov I, Rastogi N, Sola C (2002) A data-mining approach to spacer oligonucleotide typing of Mycobacterium tuberculosis. Bioinformatics 18: 235–243. 11847071 10.1093/bioinformatics/18.2.235

[66] Bouckaert RR (2010) DensiTree: making sense of sets of phylogenetic trees. Bioinformatics 26: 1372–1373. 10.1093/bioinformatics/btq110 20228129

[67] Amarreh I, Meyerand ME, Stafstrom C, Hermann BP, Birn RM (2014) Individual classification of children with epilepsy using support vector machine with multiple indices of diffusion tensor imaging. Neuroimage Clin 4: 757–764. 10.1016/j.nicl.2014.02.006 24936426 PMC4053650

[68] Ghorbani M, Taylor SJ, Pook MA, Payne A (2013) Comparative (computational) analysis of the DNA methylation status of trinucleotide repeat expansion diseases. J Nucleic Acids 2013: 689798 10.1155/2013/689798 24455203 PMC3884633

[69] Sloot R, Borgdorff MW, de Beer JL, van Ingen J, Supply P, van Soolingen D. (2013) Clustering of tuberculosis cases based on variable-number tandem-repeat typing in relation to the population structure of Mycobacterium tuberculosis in the Netherlands. J Clin Microbiol 51: 2427–2431. 10.1128/JCM.00489-13 23658260 PMC3697710

[70] van Soolingen D, Hermans PWM, de Haas PEW, Sool DR, van Embden JDA (1991) The occurence and stability of insertion sequences in *Mycobacterium tuberculosis* complex strains: evaluation of an insertion sequence-dependent DNA polymorphism as a tool in the epidemiology of tuberculosis. J Clin Microbiol 29: 2578–2586. 1685494 10.1128/jcm.29.11.2578-2586.1991 PMC270376

[71] Cowan LS, Diem L, Brake MC, Crawford JT (2004) Transfer of a Mycobacterium tuberculosis genotyping method, Spoligotyping, from a reverse line-blot hybridization, membrane-based assay to the Luminex multianalyte profiling system. J Clin Microbiol 42: 474–477. 14715809 10.1128/JCM.42.1.474-477.2004 PMC321738

[72] Zhang J, Abadia E, Refregier G, Tafaj S, Boschiroli ML, Guillard B, et al (2010) Mycobacterium tuberculosis complex CRISPR genotyping: improving efficiency, throughput and discriminative power of 'spoligotyping' with new spacers and a microbead-based hybridization assay. J Med Microbiol 59: 285–294. 10.1099/jmm.0.016949-0 19959631

[73] Filliol I, Driscoll JR, Van Soolingen D, Kreiswirth BN, Kremer K, Valétudie G, et al (2002) Global distribution of *Mycobacterium tuberculosis* spoligotypes. Emerg Inf Dis 8: 1347–1350. 10.3201/eid0811.020125 PMC2738532 12453368

[74] Filliol I, Ferdinand S, Negroni L, Sola C, Rastogi N (2000) Molecular typing of *Mycobacterium tuberculosis* based on variable number of tandem DNA repeats (VNTR) used alone, and in association with spoligotyping. J Clin Microbiol 38: 2520–2524. 10878036 10.1128/jcm.38.7.2520-2524.2000 PMC86957

[75] WHO (2006) Global Tuberculosis Control: Surveillance, Planning, Financing.: WHO, Geneva, Switzerland. WHO/CDS/TB/2002.295 WHO/CDS/TB/2002.295.

[76] de Beer JL, van Ingen J, de Vries G, Erkens C, Sebek M, Mulder A, et al (2013) Comparative study of IS6110 restriction fragment length polymorphism and variable-number tandem-repeat typing of Mycobacterium tuberculosis isolates in the Netherlands, based on a 5-year nationwide survey. J Clin Microbiol 51: 1193–1198. 10.1128/JCM.03061-12 23363841 PMC3666783

[77] Murray M (2002) Sampling bias in the molecular epidemiology of tuberculosis. Emerg Infect Dis 8: 363–369. 11971768 10.3201/eid0804.000444 PMC2730247

[78] Walker TM, Monk P, Smith EG, Peto TE (2013) Contact investigations for outbreaks of Mycobacterium tuberculosis: advances through whole genome sequencing. Clin Microbiol Infect 19: 796–802. 10.1111/1469-0691.12183 23432709

[79] Small PM, Hopewell PC, Singh SP, Paz A, Parsonnet J, Mickelsen PA (1994a) The Epidemiology of Tuberculosis in San Francisco. N Engl J Med 330: 1703–1709. 7910661 10.1056/NEJM199406163302402

[80] Lawson L, Zhang J, Gomgnimbou MK, Abdurrahman ST, Le Moullec S, Uzoewulu GN, et al (2012) A molecular epidemiological and genetic diversity study of tuberculosis in ibadan, nnewi and abuja, Nigeria. PLoS One 7: e38409 10.1371/journal.pone.0038409 22723859 PMC3377642

[81] Mokrousov I, Vyazovaya A, Narvskaya O (2014) Mycobacterium tuberculosis Latin American-Mediterranean family and its sublineages in the light of robust evolutionary markers. J Bacteriol 196: 1833–1841. 10.1128/JB.01485-13 24584500 PMC4011003

[82] Niobe-Eyangoh SN, Kuaban C, Sorlin P, Cunin P, Thonnon J, Sola C, et al (2003) Genetic biodiversity of Mycobacterium tuberculosis complex strains from patients with pulmonary tuberculosis in Cameroon. J Clin Microbiol 41: 2547–2553. 12791879 10.1128/JCM.41.6.2547-2553.2003 PMC156567

[83] Niobe-Eyangoh SN, Kuaban C, Sorlin P, Thonnon J, Vincent V, Gutierrez MC (2004) Molecular characteristics of strains of the cameroon family, the major group of Mycobacterium tuberculosis in a country with a high prevalence of tuberculosis. J Clin Microbiol 42: 5029–5035. 15528691 10.1128/JCM.42.11.5029-5035.2004 PMC525220

[84] Fenner L, Malla B, Ninet B, Dubuis O, Stucki D, Borrell S, et al (2011) "Pseudo-Beijing": evidence for convergent evolution in the direct repeat region of Mycobacterium tuberculosis. PLoS One 6: e24737 10.1371/journal.pone.0024737 21935448 PMC3172296

[85] Lan NTN, Lien HTK, Tung LB, Borgdorff MW, Kremer K, van Soolingen (2003) Mycobacterium tuberculosis Beijing genotype and risk for treatment failure and relapse, Vietnam. Emerg Infect Dis 9: 1633–1635. 14720411 10.3201/eid0912.030169 PMC3034339

[86] Borgdorff MW, van der Werf MJ, de Haas PE, Kremer K, van Soolingen D (2005) Tuberculosis elimination in the Netherlands. Emerg Infect Dis 11: 597–602. 15829200 10.3201/eid1104.041103 PMC3320334

