# Supplementary material for: Genomics and Machine Learning for Taxonomy Consensus: The Mycobacterium tuberculosis Complex Paradigm
Source: PLoS One. 2015 Jul 8;10(7):e0130912. doi: 10.1371/journal.pone.0130912 (PMC4496040; doi:10.1371/journal.pone.0130912)
Supplement: S2 Table — (DOCX) [file pone.0130912.s003.docx]

| **Genotyping method** | **Year** | **2004** | **2005** | **2006** | **2007** | **2008** | ***Sum 2004- 2008*** | **Global** |
| --- | --- | --- | --- | --- | --- | --- | --- | --- |
| Spoligo | Number of  isolates | 734 | 817 | 612 | 624 | 645 | *3432* | 3432 |
|  | Number of clusters | 84 | 92 | 78 | 78 | 88 | *420* | 279 |
|  | Total number  of clustered isolates | 564 | 614 | 434 | 455 | 476 | *2543* | 2934 |
|  | Number of unique strains | 170 | 203 | 178 | 169 | 169 | *889* | 520 |
| 24-VNTR | Number of  isolates | 741 | 819 | 613 | 632 | 649 | *3454* | 3454 |
|  | Number of clusters | 67 | 77 | 53 | 41 | 54 | *292* | 359 |
|  | Number of unique isolates | 529 | 524 | 458 | 523 | 493 | *2527* | 2092 |
|  | Total number  of clustered isolates | 212 | 295 | 155 | 109 | 156 | *927* | 1362 |
|  | RTI_(n-1)_ VNTR | 0.196 | 0.267 | 0.166 | 0.108 | 0.157 | *-* | 0.290 |
