# Supplementary material for: Genomics and Machine Learning for Taxonomy Consensus: The Mycobacterium tuberculosis Complex Paradigm
Source: PLoS One. 2015 Jul 8;10(7):e0130912. doi: 10.1371/journal.pone.0130912 (PMC4496040; doi:10.1371/journal.pone.0130912)
Supplement: Supplementary file 3 [file pone.0130912.s001.docx]

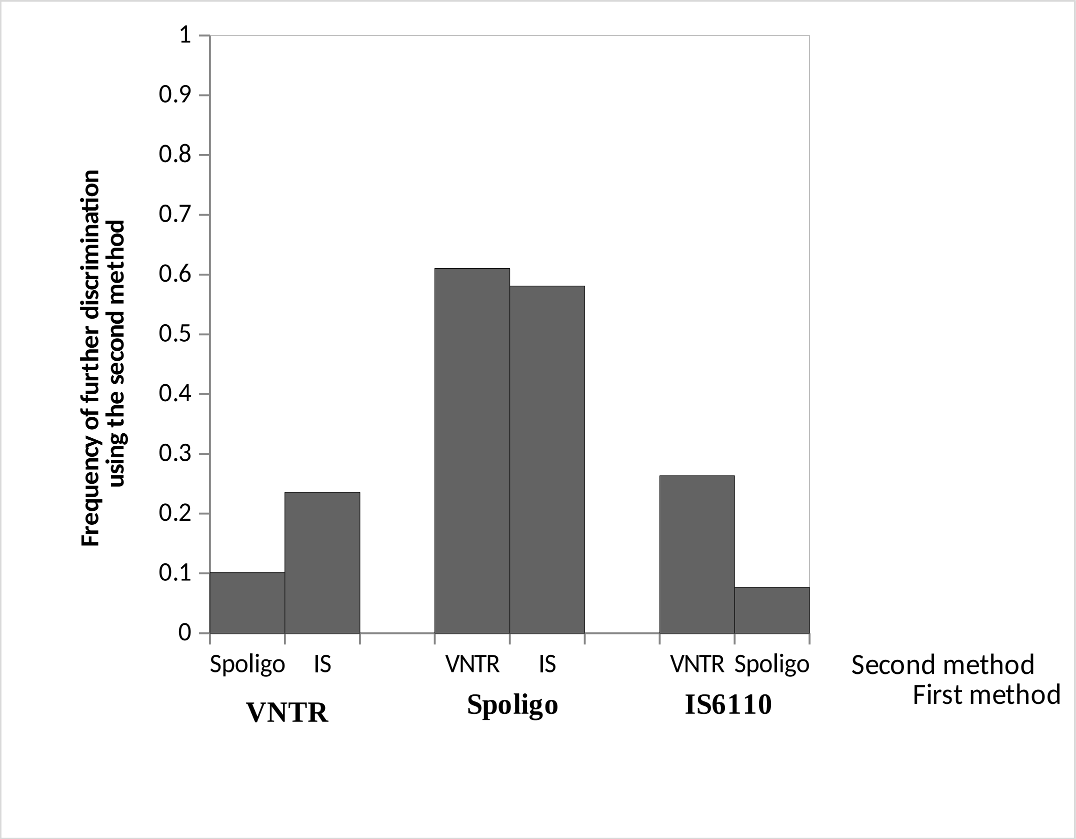

Frequency of further discrimination using the second method
1
0.9
0.8
0.7
0.6
0.5
0.4
0.3
0.2
0.1
0
Spoligo
IS
VNTR
IS
VNTR
Spoligo
Second method
First method
VNTR
Spoligo
IS6110
